# Representations for complex numbers with integer digits

**DOI:** 10.1007/s40993-020-00214-0

**Published:** 2020-11-10

**Authors:** Paul Surer

**Affiliations:** Institut für Mathematik, Universität für Bodenkultur, Gregor-Mendel-Straße 33, A-1180 Vienna, Austria

**Keywords:** Complex expansions, Radix representations, 11A63, 11B85, 11R06

## Abstract

We present the zeta-expansion as a complex version of the well-known beta-expansion. It allows us to expand complex numbers with respect to a complex base by using integer digits. Our concepts fits into the framework of the recently published rotational beta-expansions. But we also establish relations with piecewise affine maps of the torus and with shift radix systems.

## Introduction

Let $\zeta \in {{\mathbb {C}}}$ be non-real, fix an $\varepsilon \in [0,1)$, and define

$$\begin{aligned} D := \{-\overline{\zeta }\mu _1 + \mu _2 : \mu _1, \mu _2 \in [-\varepsilon , 1-\varepsilon )\} \subset {{\mathbb {C}}}. \end{aligned}$$

**Fig. 1:**
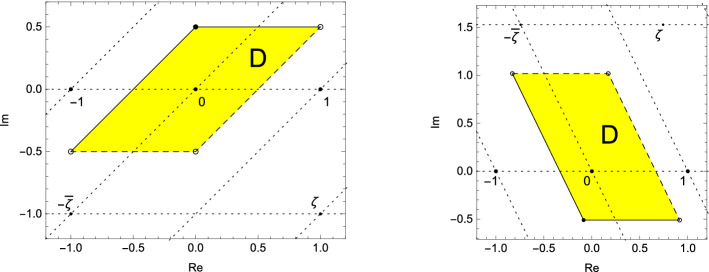
The shape and the position of the fundamental domain *D* and the lattice ${{\mathcal {L}}}_\zeta$ for two different examples. Left we chose $\zeta =1-i$ and $\varepsilon =\nicefrac {1}{2}$. Here $\zeta$ is a lattice point. On the right we have the situation for $\zeta$ one of the dominant roots of $t^4 - 2 t^3 + 4 t^2 - 2 t + 1$ (in particular, $\zeta \approx 0.7429 - 1.5291 i$ is a complex Pisot number) and $\varepsilon =\nicefrac {1}{3}$. We see that here $\zeta \not \in {{\mathcal {L}}}_\zeta$

The set *D* has the shape of a parallelepiped and is a fundamental domain for the lattice ${{\mathcal {L}}}_\zeta$ generated by $-\overline{\zeta }$ and 1. In Fig. 1 we see two different examples. We define the zeta-transformation on *D* by

$$\begin{aligned} S: D \longrightarrow D, z \longmapsto \zeta \cdot z \, (\mathrm{mod\,}{{\mathcal {L}}}_\zeta ). \end{aligned}$$

In the present article we mainly concentrate on the case that $\left|\zeta \right|>1$ and we are interested in radix representations of (complex) numbers induced by the zeta-transformation. Indeed, provided that $\left|\zeta \right|>1$ we obtain for each $z \in D$ by successive application of *S* an expansion with respect to the base $\zeta$

$$\begin{aligned} z = \sum _{n\ge 1} d_n \zeta ^{-n} \text { with } \qquad d_n = \zeta S^{n-1}(z) - S^{n}(z) \in {{\mathcal {L}}}_\zeta . \end{aligned}$$

**Fig. 2:**
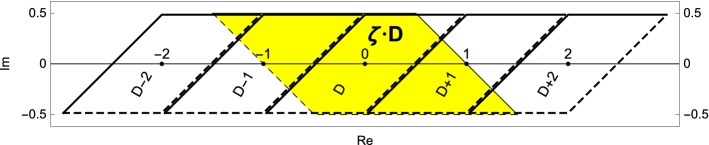
We see that $\zeta D$ (yellow) is completely covered by integer translates of the fundamental domain *D*. In the concrete example we have $\varepsilon =\nicefrac {1}{2}$ and $\zeta =1-i$. The set of digits in this example consists of the integers from $-2$ to 2

The setting fits into the framework of rotational beta-expansions recently introduced in [3, 4] as a way to generalise the real (one-dimensional) beta-expansion to higher dimensions where the focus is set on ergodicity, soficness and invariant probability measures. Therefore, we will not discuss these topics here but refer to [3, 4]. We rather concentrate on the special feature of the zeta-transformation, namely, that, due to the particular shape of our domain *D*, the produced digit sequences consist of integers only. Indeed, in Fig. 2 we see that integer translates of *D* completely cover $\zeta D$. The actual set of digits is given by

$$\begin{aligned} {{\mathcal {N}}}= {\left\{ \begin{array}{ll} \big ((\varepsilon -1) \left|\zeta -1\right|^2-2\mathrm{Re}\left( \zeta \right) , \varepsilon \left|\zeta -1\right|^2+ 2\mathrm{Re}\left( \zeta \right) \big ) \cap {{\mathbb {Z}}}&{} \text {if } \mathrm{Re}\left( \zeta \right) > 0, \\ \big ((\varepsilon -1)\left|\zeta -1\right|^2, \varepsilon \left|\zeta -1\right|^2\big ] \cap {{\mathbb {Z}}}&{} \text {if } \mathrm{Re}\left( \zeta \right) \le 0. \\ \end{array}\right. } \end{aligned}$$

We will characterise the admissible sequences in terms of real numbers by Dickson polynomials of the second type which are generalisations of Chebychev polynomials (see [40]). In general the digit sequences produced by *S* have a quite complicated structure. Indeed, from [4] we conclude that the induced shift spaces are rarely sofic. A necessary condition is that the argument of $\zeta$ is a rational multiple of $\pi$.

**Fig. 3:**
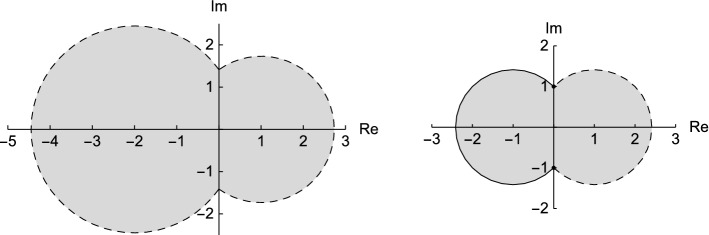
The bases outside the grey area provide unique zeta-expansions. On the left we see the case $\varepsilon =\nicefrac {1}{3}$. The case $\varepsilon =\nicefrac {1}{2}$ on the right is the most universal case, that is the bases in the grey area do not provide unique zeta-expansions for any choice of $\varepsilon$

A priori the zeta-transformation allows us to represent the elements of *D* only. By the *zeta-expansion* we mean the extension to the entire complex plane. We ask for the uniqueness of this extension (up to leading zeros) and we will see that the zeta-expansion is unique for bases that have sufficiently large distance from 1 and $-\max \{(1-\varepsilon )\varepsilon ^{-1},\varepsilon (1-\varepsilon )^{-1}\}$. Especially, if $\varepsilon =0$ then the zeta-expansion is not unique for any base. Figure 3 shows the set of bases that do not provide a unique zeta-expansion for two different choices of $\varepsilon$.

Complex expansions with respect to imaginary bases are intimately related with real expansions. Indeed, this case can easily be reduced to a separate expansion of the real part and the imaginary part (see [35]). We are going to observe a similar behaviour for integer multiples of a third, sixth or eighth root of unity. An intensive discussion of such bases will exhibit quite interesting properties of zeta-expansions of complex numbers.

Finally we are concerned with bases $\zeta$ that are algebraic integers. We will see that the behaviour of the zeta-transformation *S* on the elements of ${{\mathbb {Z}}}[\zeta ]$ can be described with the aid of shift radix systems (see [2]). In fact, our result is quite analogous to the well-known relation between shift radix systems and beta-expansions with respect to algebraic integer.

There exist several further approaches for complex expansions. Pioneering work was performed in [46] and [35]. Gilbert observed in [24] that all complex numbers allow a radix representation with respect to the base $-m\pm i$ ($m \in {{\mathbb {N}}}$) and set of digits $\{0, \ldots , m^2\}$. He also observed that the fundamental domain, that is the set of numbers with integer part zero, has a fractal shape (see also [35]). This fact makes it difficult to identify the elements with two or three representations (see [23, 25]). In other words, there is no straightforward analogue to the well-known decimal relation $0.99\cdots = 1.00\cdots$. In general it is also difficult to explicitly determine a representation for a given complex number (that is not contained in ${{\mathbb {Z}}}[\zeta ^{-1}]$).

Gilbert’s considerations fit into the framework of Canonical number systems (see [33, 38, 39, 47, 53]) that all have the mentioned characteristics: comfortable digit strings (the full shift) but problems in expanding arbitrary numbers and characterising the elements with two or even more expansions. Furthermore, not all bases induce a Canonical number system and the characterisation of respective bases is up to now a challenge. An overview can be obtained from the quite recent article [48] and the references within. This exhibits the differences to the present approach: it is defined via an explicit algorithm for obtaining the expansions and the fundamental domain has a simple structure. However, as already mentioned the digit strings are quite difficult to characterise. We will explicitly compare our expansion with Canonical number system (with respect to the same base) by an example.

Another way to obtain complex expansions is presented in [8, 21, 27] where the bases are complex Pisot numbers, more precisely, complex Pisot units, hence, complex conjugate pairs of algebraic units such that the other Galois conjugates are inside the unit circle. This type of numeration system is derived from the theory of unimodular Pisot substitutions and Rauzy fractals, hence, again uniqueness is a difficult task due to the fractal structures. Further - and more exceptional - ideas for complex expansions can be found in [13, 36, 50].

We also want to mention the articles [17, 19, 20, 43, 44] that deal with on-line algorithms for algebraic operations in complex positional representations without considering the question of (unique) representability.

If $\zeta$ is located on the unit circle then *S* corresponds to a piecewise isometry on the torus. The dynamical behaviour of such maps has been intensively studied, for example, in [1, 12, 37, 59]. There are also applications in signal precessing (second order digital filters, see, for example, [14, 60, 61]). A more detailed overview on this huge topic can be obtained from [41] and the references therein.

The article is organised in the following way. In Sect. 2 we properly state all necessary definitions and describe the zeta-transformation in terms of a piecewise affine map of the torus. This will allow us to show that the set of digits ${{\mathcal {N}}}$ has the stated shape and to describe the admissible sequences by Dickson polynomials of the second kind. In Sect. 3 we investigate whether it is possible to uniquely extend the expansions to the entire complex plane (zeta-expansion). In Sect. 4 we show that for special choices of $\zeta$ the zeta-transformation is related with certain real transformations. In this case we can describe the admissible sequences by inequalities with respect to the lexicographical order or the alternating order. In Sect. 5 we establish the relation with shift radix systems and discuss finiteness and periodicity properties.

## The zeta-transformation and its basic properties

In the present section we state all necessary definitions and notations in a proper way and we characterise the zeta-transformation in terms of a piecewise affine map of the torus.

Fix an $\varepsilon \in [0,1)$ and define $I_\varepsilon =[-\varepsilon , 1-\varepsilon )$. Let $\zeta \in {{\mathbb {C}}}\setminus {{\mathbb {R}}}$ and

$$\begin{aligned} D=D_{\zeta ,\varepsilon }:=\{-\overline{\zeta }\mu _1 + \mu _2 : (\mu _1, \mu _2) \in I_\varepsilon ^2\} \subset {{\mathbb {C}}}. \end{aligned}$$

Note that actually all the notations in this article depend on $\zeta$ or $\varepsilon$ and we mark this by lower indices. For convenience we will frequently skip these indices when there is no danger of confusion.

The set *D* is a fundamental domain of the lattice ${{\mathcal {L}}}_\zeta$ generated by $-\overline{\zeta }$ and 1. We are interested in the transformation

$$\begin{aligned} S=S_{\zeta ,\varepsilon }: D \longrightarrow D, z \longmapsto \zeta z - d \end{aligned}$$

where $d \in {{\mathcal {L}}}_\zeta$ is the uniquely determined lattice point such $\zeta z -d \in D$. We call the transformation *S* the zeta-transformation.

For $z \in D$ we define the digit sequence $\mathbf {d}_{\zeta ,\varepsilon }(z) := (d_n)_{n \ge 1}$ obtained from the successive application of *S*, hence, $d_n = \zeta S^{n-1}(z)- S^n(z)$. Obviously,

2.1 $$\begin{aligned} z=\sum _{n \ge 1} d_n \zeta ^{-n}. \end{aligned}$$

Let us fix some useful notations. We denote by $\left\lfloor \cdot \right\rfloor _\varepsilon$ the generalised floor function that is defined for real numbers *x* by

$$\begin{aligned} \left\lfloor x \right\rfloor _\varepsilon = \left\lfloor x+\varepsilon \right\rfloor , \end{aligned}$$

where $\left\lfloor x \right\rfloor$ is the usual floor function, i.e. the largest integer smaller than or equal to *x*. Analogously, we write $\left\{ \cdot \right\} _\varepsilon$ for the respective generalised fractional part, hence,

$$\begin{aligned} \left\{ x\right\} _\varepsilon = x - \left\lfloor x \right\rfloor _\varepsilon \in I_\varepsilon . \end{aligned}$$

For real parameters $a_0,a_1$ we define the transformation

$$\begin{aligned} {\varvec{A}}_{a_0,a_1}: I_\varepsilon ^2 \longrightarrow I_\varepsilon ^2, (\mu _1, \mu _2) \longmapsto \big (\mu _2, \left\{ a_0 \mu _1 + a_1 \mu _2\right\} _\varepsilon \big ). \end{aligned}$$

For a special choice of $a_0, a_1$ the transformation ${\varvec{A}}_{a_0,a_1}$ is intimately related with the zeta-transformation *S* via the (bijective) map

$$\begin{aligned} {\varvec{\psi }}_\zeta : {{\mathbb {C}}}\longrightarrow {{\mathbb {R}}}^2, z \longmapsto \left( \frac{z-{\overline{z}}}{\zeta -\overline{\zeta }}, \frac{\zeta z-\overline{\zeta z}}{\zeta -\overline{\zeta }} \right) . \end{aligned}$$

### Proposition 1

For any $\zeta \in {{\mathbb {C}}}\setminus {{\mathbb {R}}}$ the function ${\varvec{\psi }}_\zeta$ is a bijection and for each $z \in D$ we have

$$\begin{aligned} {\varvec{A}}_{a_0,a_1} \circ {\varvec{\psi }}_\zeta (z)= {\varvec{\psi }}_\zeta \circ S (z) \end{aligned}$$

with $$a_0=-\left|\zeta \right|^2= -\zeta \overline{\zeta }$$ and $$a_1=2\mathrm{Re}\left( \zeta \right) = \zeta + \overline{\zeta }$$.

### Proof

Let us consider the complex plane as two-dimensional real vector space. Then $\{-\overline{\zeta }, 1\}$ provides a basis since $\zeta \not \in {{\mathbb {R}}}$. Hence, for each $z \in {{\mathbb {C}}}$ there is a uniquely determined vector $(\mu _0, \mu _1) \in {{\mathbb {R}}}^2$ such that $z=\mu _0(-\overline{\zeta }) +\mu _1$. One readily verifies that, indeed, we have $(\mu _0, \mu _1) = {\varvec{\psi }}_\zeta (z)$.

In order to show that the transformations *S* and ${\varvec{A}}_{a_0,a_1}$ commute via ${\varvec{\psi }}_\zeta$ let $z \in D$ and note that this is equivalent to ${\varvec{\psi }}_\zeta (z) \in I_\varepsilon ^2$. We obtain

$$\begin{aligned} \zeta \cdot z = \zeta (\mu _0(-\overline{\zeta }) +\mu _1) = \mu _1 (-\overline{\zeta }) + (-\zeta \overline{\zeta } \mu _0 + (\zeta +\overline{\zeta }) \mu _1) = \mu _1 (-\overline{\zeta }) + (a_0\mu _0 + a_1 \mu _1). \end{aligned}$$

Set $d:=\left\lfloor a_0\mu _0 + a_1 \mu _1 \right\rfloor _\varepsilon$ and observe that

$$\begin{aligned} \zeta \cdot z - d = \mu _1 (-\overline{\zeta }) + \left\{ a_0\mu _0 + a_1 \mu _1\right\} _\varepsilon \in D. \end{aligned}$$

Since $d\in {{\mathbb {Z}}}$ is a lattice point of the lattice ${{\mathcal {L}}}_\zeta$ we conclude that $S(z)=\mu _1 (-\overline{\zeta }) + \left\{ a_0\mu _0 + a_1 \mu _1\right\} _\varepsilon$ and obviously ${\varvec{\psi }}_\zeta \circ S (z) = (\mu _1 , \left\{ a_0\mu _0 + a_1 \mu _1\right\} _\varepsilon )$. On the other hand, by the definition of ${\varvec{A}}_{a_0,a_1}$ we have ${\varvec{A}}_{a_0,a_1} \circ {\varvec{\psi }}_\zeta (z) = (\mu _1, \left\{ a_0\mu _0 + a_1 \mu _1\right\} _\varepsilon )$. $\square$

Note that $\zeta$ and $\overline{\zeta }$ are the roots of the real polynomial $t^2-a_1t-a_0$. The assertion of the proposition can be visualised by the following commutative diagram.

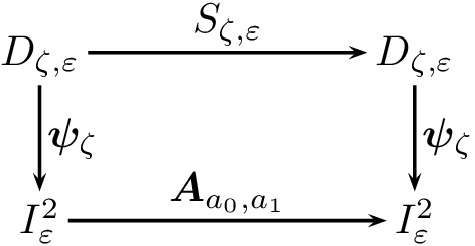



With this result we can state the zeta-transformation in the following way.

### Corollary 1

For all $z \in D$ we have

$$\begin{aligned} S(z)= \zeta z -\left\lfloor \frac{\zeta ^2 z-\overline{\zeta ^2 z}}{\zeta -\overline{\zeta }} \right\rfloor _\varepsilon . \end{aligned}$$

### Proof

From the proof of Proposition 1 we see that $S(z)= \zeta z -d$ with $d= \left\lfloor a_0 \mu _0 + a_1 \mu _1 \right\rfloor _\varepsilon$, $a_0=-\zeta \overline{\zeta }$, $a_1=\zeta + \overline{\zeta }$, and $(\mu _0,\mu _1)={\varvec{\psi }}_\zeta (z)$. Inserting this immediately yields

$$\begin{aligned} d=\left\lfloor -\zeta \overline{\zeta }\frac{z-{\overline{z}}}{\zeta -\overline{\zeta }} +(\zeta + \overline{\zeta }) \frac{\zeta z-\overline{\zeta z}}{\zeta -\overline{\zeta }} \right\rfloor _\varepsilon = \left\lfloor \frac{\zeta ^2 z-\overline{\zeta ^2 z}}{\zeta -\overline{\zeta }} \right\rfloor _\varepsilon . \end{aligned}$$

$$\square $$

From this we also see that $\mathbf {d}_{\zeta ,\varepsilon }(z)$ is an integer sequence. The exact shape of the set of digits is stated in the next corollary.

### Corollary 2

For each $z \in D$ we have $\mathbf {d}_{\zeta ,\varepsilon }\in {{\mathcal {N}}}^{{\mathbb {N}}}$ with

$$\begin{aligned} {{\mathcal {N}}}={{\mathcal {N}}}_{\zeta ,\varepsilon }:= {\left\{ \begin{array}{ll} \big ((\varepsilon -1) \left|\zeta -1\right|^2-2\mathrm{Re}\left( \zeta \right) , \varepsilon \left|\zeta -1\right|^2+2\mathrm{Re}\left( \zeta \right) )\big ) \cap {{\mathbb {Z}}}&{} \text {if } \mathrm{Re}\left( \zeta \right) > 0, \\ \big ((\varepsilon -1)\left|\zeta -1\right|^2, \varepsilon \left|\zeta -1\right|^2\big ] \cap {{\mathbb {Z}}}&{} \text {if } \mathrm{Re}\left( \zeta \right) \le 0. \\ \end{array}\right. } \end{aligned}$$

### Proof

For each digit *d* we have $d=\left\lfloor a_0 \mu _0 + a_1 \mu _1 \right\rfloor _\varepsilon$ and, hence, $-\varepsilon \le a_0 \mu _0 + a_1 \mu _1 - d <1- \varepsilon$ where $a_0=-\left|\zeta \right|^2 <0$, $a_1= 2\mathrm{Re}\left( \zeta \right)$, and $-\varepsilon \le \mu _0, \mu _1 < 1-\varepsilon$. From this we easily obtain the statement by observing that $\left|\zeta -1\right|^2 =1-2\mathrm{Re}\left( \zeta \right) +\left|\zeta \right|^2 = 1-a_1-a_0$. $\square$

Note that the set of digits ${{\mathcal {N}}}$ contains 0, independently of the choice of $\zeta$ and $\varepsilon$.

### Example 1

Let $\zeta =3 e^{\nicefrac {2\pi i}{3}}=-\nicefrac {3}{2}+\nicefrac {3\sqrt{3}i}{2}$ and $\varepsilon =\nicefrac {1}{2}$. Proposition 1 provides a convenient way to obtain $\mathbf {d}_{\zeta ,\varepsilon }(z)$ for a given $z \in D$. For this reason we set $a_0:=-\left|\zeta \right|^2=-9$ and $a_1:=2\mathrm{Re}\left( \zeta \right) =-3$. Observe that the real part of $\zeta$ is negative. Therefore, the set of digits is given by ${{\mathcal {N}}}:=\{-6, \ldots , 6\}$. Table 1 shows $\mathbf {d}_{\zeta ,\varepsilon }(z)$ for several choices of *z*.

**Table 1:** Digit sequences $\mathbf {d}_{\zeta ,\varepsilon }(z)$ for different choices of *z*. Here $\zeta =3 e^{\nicefrac {2\pi i}{3}}$ and $\varepsilon =\nicefrac {1}{2}$ (see Example 1)

*z*	${\varvec{\psi }}_{\zeta }(z)$	$\mathbf {d}_{\zeta ,\varepsilon }(z)$
$\nicefrac {1}{3}$	$(0,\nicefrac {1}{3})$	$-1,-3,(0)^\omega$
$\nicefrac {\overline{\zeta }}{3}$	$(-\nicefrac {1}{3},0)$	$3,(0)^\omega$
$\nicefrac {-\zeta }{9}$	$(-\nicefrac {1}{9},\nicefrac {1}{3})$	$0,-3,(0)^\omega$
$\left( \zeta -1\right) ^{-1}$	$(-\nicefrac {1}{13},-\nicefrac {1}{13})$	$(1)^\omega$
$6\left( 1-\zeta \right) ^{-1}$	$(\nicefrac {6}{13},\nicefrac {6}{13})$	$(-6)^\omega$
$\left( \zeta +1\right) ^{-1}$	$(-\nicefrac {1}{7},\nicefrac {1}{7})$	$(1,-1)^\omega$
$3\left( -\zeta -1\right) ^{-1}$	$(\nicefrac {3}{7},-\nicefrac {3}{7})$	$(3,-3)^\omega$
$-\nicefrac {1}{2}$	$(0,-\nicefrac {1}{2})$	$(2,6,5)^\omega$
$\nicefrac {\overline{\zeta }}{2}$	$(-\nicefrac {1}{2},0)$	$(5,2,6)^\omega$
$\nicefrac {(\overline{\zeta }-1)}{2}$	$(-\nicefrac {1}{2},-\nicefrac {1}{2})$	$(6,5,2)^\omega$
$-\nicefrac {\overline{\zeta }}{4}-\nicefrac {1}{3}$	$(\nicefrac {1}{4},-\nicefrac {1}{3})$	$-1,4,(3,2,-1,-3,-2,1)^\omega$

### Example 2

Let $\zeta =\sqrt{2} e^{-\nicefrac {\pi i}{4}}=1-i$ and $\varepsilon =\nicefrac {1}{2}$. We set $a_0:=-\left|\zeta \right|^2=-2$ and $a_1:=2\mathrm{Re}\left( \zeta \right) =2$. In this example the real part of the base $\zeta$ is positive, the set of digits here is given by ${{\mathcal {N}}}:=\{-2, \ldots , 2\}$. The situation is sketched on the left hand side of Fig. 1 and in Fig. 2. In Table 2 we calculate $\mathbf {d}_{\zeta ,\varepsilon }(z)$ for different choices of *z*.

**Table 2:** Digit sequences $\mathbf {d}_{\zeta ,\varepsilon }(z)$ for different choices of *z*. Here $\zeta =1-i$ and $\varepsilon =\nicefrac {1}{2}$ (see Example 2)

*z*	${\varvec{\psi }}_{\zeta }(z)$	$\mathbf {d}_{\zeta ,\varepsilon }(z)$
$\nicefrac {3}{4}+\nicefrac {i}{2}$	$(-\nicefrac {1}{2},\nicefrac {1}{4})$	$2,-1,0,1,(0)^\omega$
$-\nicefrac {5}{16}+\nicefrac {i}{16}$	$(-\nicefrac {1}{16},-\nicefrac {3}{8})$	$-1,2,-2,2,-1,0,1,(0)^\omega$
$-\nicefrac {1}{2}$	$(0,-\nicefrac {1}{2})$	$-1,1,(0)^\omega$
$\nicefrac {i}{2}$	$(-\nicefrac {1}{2},-\nicefrac {1}{2})$	$0,1,(0)^\omega$
$\nicefrac {1}{2} - \nicefrac {i}{2}$	$(-\nicefrac {1}{2},0)$	$1,(0)^\omega$
$\nicefrac {2}{5}+\nicefrac {i}{5}$	$(-\nicefrac {1}{5},\nicefrac {1}{5})$	$(1,-1)^\omega$
$-\nicefrac {4}{5}-\nicefrac {2i}{5}$	$(\nicefrac {2}{5},-\nicefrac {2}{5})$	$(-2,2)^\omega$
$-\nicefrac {3}{10}-\nicefrac {i}{10}$	$(\nicefrac {1}{10},-\nicefrac {1}{5})$	$-1,(1,0,-1,0)^\omega$
$-\nicefrac {2}{5}-\nicefrac {i}{3}$	$(\nicefrac {1}{3},-\nicefrac {1}{15})$	$(-1,1,-1,0,1,0,-1,1)^\omega$

### Definition 1

Let $\zeta \in {{\mathbb {C}}}\setminus {{\mathbb {R}}}$ and $\varepsilon \in [0,1)$. A sequence $(d_n)_{n \ge 1} \in {{\mathbb {Z}}}^\infty$ is called (${\zeta ,\varepsilon }$)-admissible if there exists a $z \in D$ such that $\mathbf {d}_{\zeta ,\varepsilon }(z)=(d_n)_{n \ge 1}$. The set

$$\begin{aligned} \Omega _{\zeta ,\varepsilon }:= \overline{\{\mathbf {d}_{\zeta ,\varepsilon }(z) : z \in D\}} \end{aligned}$$

(the closure with respect to the product topology of the discrete topology) is the corresponding *zeta-shift*.

Define for each (positive and negative) integer *n* the expression

$$\begin{aligned} P_n(\zeta ):=\frac{\zeta ^{n} - \overline{\zeta ^{n}}}{\zeta - \overline{\zeta }} \in {{\mathbb {R}}}. \end{aligned}$$

Observe that $P_n(\zeta )= -(\zeta \overline{\zeta })^{n}P_{-n}(\zeta )$ holds for all $n \in {{\mathbb {Z}}}$. Furthermore, the recursion formula

2.2 $$\begin{aligned} P_n(\zeta ) = 2\mathrm{Re}\left( \zeta \right) P_{n-1}(\zeta )-\left|\zeta \right|^2P_{n-2}(\zeta ) \end{aligned}$$

is satisfied. We have $P_0(\zeta )=0$, $P_1(\zeta )=1$, $P_2(\zeta )=2\mathrm{Re}\left( \zeta \right)$, and $P_{n+1}(\zeta )=E_{n}(2\mathrm{Re}\left( \zeta \right) , \left|\zeta \right|^2) = \left|\zeta \right|^nU_n(\mathrm{Re}\left( \zeta \right) \left|\zeta \right|^{-1})$ for all $n \ge 0$, where $E_{n}$ denotes the *n*th Dickson polynomial of the second kind and $U_n$ denotes the *n*th Chebyshev polynomial of the second kind. Details on Dickson polynomials can be found in [40].

### Theorem 1

Let $\zeta \in {{\mathbb {C}}}\setminus {{\mathbb {R}}}$ with $\left|\zeta \right|>1$, $\varepsilon \in [0,1)$ and $(d_n)_{n \ge 1}$ an integer sequence. Then the following items are equivalent:

(i) $$(d_n)_{n \ge 1} \in {{\mathbb {Z}}}^{{\mathbb {N}}}$$ is $$({\zeta ,\varepsilon })$$-admissible;
(ii) $\sum _{n \ge 1} d_{n+k}\zeta ^{-n} \in D$ for all $k \ge 0$;
(iii) $\sum _{n \ge 1} d_{n+k}P_{-n}(\zeta ) \in I_\varepsilon$ for all $k \ge 0$.

### Proof

We start with (i) $\Rightarrow$ (ii) and suppose that $(d_n)_{n \ge 1}$ is $({\zeta ,\varepsilon })$-admissible. Then there exists a $z \in D$ such that $\mathbf {d}_{\zeta ,\varepsilon }(z)= (d_n)_{n \ge 1}$. By definition we have $S^k(z) = d_{k+1}\zeta ^{-1} + S^{k+1}(z)\zeta ^{-1} \in D$ for each $k \ge 0$ and since $\left|\zeta \right|>1$ this immediately yields that $S^k(z) = \sum _{n \ge 1} d_{n+k}\zeta ^{-n} \in D$.

To show that (ii) $\Rightarrow$ (iii) we apply ${\varvec{\psi }}_\zeta$ and obtain

$$\begin{aligned} {\varvec{\psi }}_\zeta (S^k(z))=&\left( \sum _{n \ge 1} d_{n+k}\frac{\zeta ^{-n}-\overline{\zeta ^{-n}}}{\zeta -\overline{\zeta }}, \sum _{n \ge 1} d_{n+k+1}\frac{\zeta ^{-n}-\overline{\zeta ^{-n}}}{\zeta -\overline{\zeta }}\right) \\ =&\left( \sum _{n \ge 1} d_{k+n}P_{-n}(\zeta ), \sum _{n \ge 1} d_{k+n+1}P_{-n}(\zeta )\right) \in I_\varepsilon ^2. \end{aligned}$$

Finally, we show that (iii) $\Rightarrow$ (i). For each $k \ge 0$ define $\mu _k:=\sum _{n \ge 1} d_{k+n}P_{-n}(\zeta )$ and $z:=-\overline{\zeta }\mu _0 + \mu _1$. Then we have for $a_0:=-\left|\zeta \right|^2$ and $a_1:=2\mathrm{Re}\left( \zeta \right)$

$$\begin{aligned} {\varvec{A}}_{a_0,a_1}(\mu _k,\mu _{k+1}) = \left( \mu _{k+1},\left\{ -\zeta \overline{\zeta } \mu _{k} +(\zeta +\overline{\zeta }) \mu _{k+1}\right\} _\varepsilon \right) . \end{aligned}$$

By observing the recursion (2.2) and since $P_0(\zeta )=0$ and $P_1(\zeta )=1$ we obtain

$$\begin{aligned}&-\zeta \overline{\zeta } \mu _{k} + (\zeta +\overline{\zeta }) \mu _{k+1} \\&\quad = -\zeta \overline{\zeta } P_{-1}(\zeta )d_{k+1} + \sum _{n \ge 1} d_{n+k+1} \big (-\zeta \overline{\zeta }P_{-n-1}(\zeta )+(\zeta +\overline{\zeta })P_{-n}(\zeta )\big ) \\&\quad = d_{k+1} + \sum _{n \ge 1} d_{n+k+1} P_{-n+1}(\zeta ) =d_{k+1} + \sum _{n \ge 1} d_{n+k+2} P_{-n}(\zeta ) = d_{k+1} + \mu _{k+2}. \end{aligned}$$

Since $\mu _{k+2} \in I_\varepsilon$ this immediately yields ${\varvec{A}}_{a_0,a_1}(\mu _k,\mu _{k+1}) = (\mu _{k+1},\mu _{k+2})$. From Proposition 1 we now see that $S^k(z)=\zeta S^{k-1}(z) + d_k$ and, hence $\mathbf {d}_{\zeta ,\varepsilon }(z)= (d_n)_{n \ge 1}$ which shows that $(d_n)_{n \ge 1}$ is $(\zeta ,\varepsilon )$-admissible. $\square$

Note that the soficness of zeta-shifts has already been studied within the more general framework discussed in [4]. It turned out that $\Omega _{\zeta ,\varepsilon }$ is not sofic for the majority of pairs $({\zeta ,\varepsilon })$. We have the following sufficient conditions for $\Omega _{\zeta ,\varepsilon }$ to be sofic.

### Proposition 2

(*cf.* [4, Theorem 1.5]) If $\left|\zeta \right|$ is a Pisot number, $\zeta \left|\zeta \right|^{-1}$ is a *q*-th root of unity and $2\mathrm{Re}\left( \zeta \right)$ as well as $\varepsilon$ are contained in ${{\mathbb {Q}}}(\left|\zeta \right|)$ then the zeta-shift $\Omega _{\zeta ,\varepsilon }$ is sofic.

## The zeta-expansion

Let $\zeta \in {{\mathbb {C}}}\setminus {{\mathbb {R}}}$ with $\left|\zeta \right|>1$. Then we can represent each $z \in D$ as

$$\begin{aligned} z = \sum _{n \ge 1} d_{n} \zeta ^{-n} \end{aligned}$$

where $\mathbf {d}_{\zeta ,\varepsilon }(z)= (d_n)_{n \ge 1}$. Now we ask whether we can extend our representations to the entire complex plane in a unique way. For this reason we require that

3.1 $$\begin{aligned} \zeta ^{-1}D \subset D. \end{aligned}$$

Observe that this condition is never fulfilled if $\varepsilon =0$. Indeed, suppose that $\varepsilon =0$ and let $z:=x \in (0,1)$ be a real number. Since $I_\varepsilon =[0,1)$ we clearly have that $z \in D$. Then, $\zeta ^{-1}z = \overline{\zeta }(\zeta \overline{\zeta })^{-1} x = -x\left|\zeta \right|^{-2} \cdot (-\overline{\zeta })$ and, hence, $\zeta ^{-1}z \not \in D$ since $-x\left|\zeta \right|^{-2} \not \in I_\varepsilon$.

The next theorem shows that Condition (3.1) suffices to uniquely represent the entire complex plane. Afterwards, in Proposition 3, we will explicitly characterise pairs $({\zeta ,\varepsilon })$ that fulfil Condition (3.1).

### Theorem 2

Let $\zeta \in {{\mathbb {C}}}\setminus {{\mathbb {R}}}$ with $\left|\zeta \right|>1$, and $\varepsilon \in [0,1)$ such that Condition (3.1) is satisfied. Then for each complex number $z \in {{\mathbb {C}}}\setminus \{0\}$ there exists an integer *m* and a $(\zeta ,\varepsilon )$-admissible sequence $(d_n)_{n \ge 1}$, both uniquely determined, that satisfy $d_1 \not =0$ and

3.2 $$\begin{aligned} z = \sum _{n \ge 1} d_n \zeta ^{-n+m}. \end{aligned}$$

### Proof

Let $z \in {{\mathbb {C}}}\setminus \{0\}$ and choose $m \in {{\mathbb {Z}}}$ such that $\zeta ^{-m} z \in D$ and $\zeta ^{-m+1} z \not \in D$. Observe that *m* is uniquely determined. Indeed, Condition (3.1) induces that there exists at most one such *m*. On the other hand, we already observed that $\varepsilon \not =0$ and, hence, 0 is located in the interior of *D*. Thus, $\left|\zeta \right|>1$ implies the existence of at least one *m* with the desired properties.

Now let $\mathbf {d}_{\zeta ,\varepsilon }(\zeta ^{-m}z) = (d_n)_{n \ge 1}$. By definition the sequence $(d_n)_{n \ge 1}$ is $({\zeta ,\varepsilon })$-admissible and satisfies (3.2). Furthermore we claim that $d_1 \not =0$. Indeed, if $\mathbf {d}_{\zeta ,\varepsilon }(\zeta ^{-m}z)$ started with 0 then we would have $S(\zeta ^{-m}z) = \zeta (\zeta ^{-m}z) - 0 = \zeta ^{-m+1}z \in D$ which contradicts the definition of *m*.

To show the uniqueness suppose there existed $m, m' \in {{\mathbb {Z}}}$ and $(\zeta ,\varepsilon )$-admissible integer sequences $(d_n)_{n \ge 1}$ and $(d'_n)_{n \ge 1}$ such that

$$\begin{aligned}\sum _{n \ge 1} d_n \zeta ^{-n+m} = z = \sum _{n \ge 1} d'_n \zeta ^{-n+m'}\end{aligned}$$

and $d_1 \not =0$ as well as $d'_1\not =0$.

The admissibility immediately implies $(d_n)_{n \ge 1} = \mathbf {d}_{\zeta ,\varepsilon }(\zeta ^{-m}z)$ and $(d'_n)_{n \ge 1} = \mathbf {d}_{\zeta ,\varepsilon }(\zeta ^{-m'}z)$. Therefore, if $m=m'$ then we necessarily have that $(d_n)_{n \ge 1} = (d'_n)_{n \ge 1}$. If $m \not =m'$ then we may assume, without loss of generality, that $m <m'$. Since $\zeta ^{-m}z \in D$ and Condition (3.1) holds we conclude that $\zeta ^{-m-k}z \in D$ for all $k \ge 1$, especially $\zeta ^{-m'+1}z \in D$. This immediately implies that $d'_1=0$ which contradicts the assumption. $\square$

Theorem 2 shows that if Condition (3.1) is satisfied then each $z \in {{\mathbb {C}}}\setminus \{0\}$ is uniquely represented by an $m \in {{\mathbb {Z}}}$ and a $(\zeta ,\varepsilon )$-admissible sequence $(d_n)_{n \ge 1}$. We call this representation the *zeta-expansion* of *z* (with respect to $({\zeta ,\varepsilon })$). and write it as

$$\begin{aligned} (z)_{\zeta ,\varepsilon }= \left\{ \begin{array} {llll} &{}0{\bullet }&{} \underbrace{0\cdots 0}_{m}d_{1}d_{2}\cdots &{} \text {if } m \ge 0, \\ d_1 d_{2}\cdots d_{-m}&{}\!\!{\bullet }\!\!\!\!&{}\!\!\!\!\!\!d_{-m+1}d_{-m+2}\cdots &{} \text {if } m < 0. \end{array}\right. \end{aligned}$$

The zeta-expansion of $z= 0$ will be simply denoted by $0\bullet$. Clearly, $z \in D$ if and only if the zeta-expansion of *z* has no integer part, i.e. it starts with $0\bullet$.

In Theorem 2 we have seen the importance of Condition (3.1) in context with unique representations. But for which choices of $\zeta$ and $\varepsilon$ is it fulfilled? We already have seen that if $\varepsilon =0$ then Condition (3.1) is not satisfied for any $\zeta$.

### Proposition 3

Let $\zeta \in {{\mathbb {C}}}\setminus {{\mathbb {R}}}$ with $\left|\zeta \right|>1$. Then Condition (3.1) is satisfied if and only if

$$\begin{aligned}\varepsilon \in {\left\{ \begin{array}{ll} \left[ \frac{1}{\left|\zeta -1\right|^2}, 1-\frac{1}{\left|\zeta -1\right|^2}\right] &{} \text {for } \mathrm{Re}\left( \zeta \right) > 0, \\ \left[ 1-\frac{\left|\zeta \right|^2}{\left|\zeta -1\right|^2}, \frac{\left|\zeta \right|^2}{\left|\zeta -1\right|^2}\right) &{} \text {for } \mathrm{Re}\left( \zeta \right) \le 0. \end{array}\right. }\end{aligned}$$

### Proof

Let $z \in D$ and $(\mu _1, \mu _2)={\varvec{\psi }}_\zeta (z) \in I_\varepsilon ^2$. Then we have $z=\mu _1 (-\overline{\zeta }) + \mu _2$ and

$$\begin{aligned} \zeta ^{-1}z = \left( \mu _1\frac{2\mathrm{Re}\left( \zeta \right) }{\left|\zeta \right|^2} - \mu _2\frac{1}{\left|\zeta \right|^2}\right) (-\overline{\zeta }) + \mu _1 \end{aligned}$$

which yields that ${\varvec{\psi }}_\zeta (\zeta ^{-1} z) =(\mu _0, \mu _1)$ with

3.3 $$\begin{aligned} \mu _0=\frac{\mu _1\cdot 2\mathrm{Re}\left( \zeta \right) - \mu _2}{\left|\zeta \right|^2}. \end{aligned}$$

Thus, $\zeta ^{-1}z \in D$ if and only if $\mu _0 \in I_\varepsilon$. Furthermore, keep in mind that

3.4 $$\begin{aligned} 1-2\mathrm{Re}\left( \zeta \right) +\left|\zeta \right|^2=\left|\zeta -1\right|^2. \end{aligned}$$

At first suppose $\varepsilon$ to be contained in the stated interval. We start with the case $\mathrm{Re}\left( \zeta \right) > 0$, hence

3.5 $$\begin{aligned} 1 \le \varepsilon \left|\zeta -1\right|^2 \le \left|\zeta -1\right|^2-1. \end{aligned}$$

Since $\mu _1, \mu _2 \in I_\varepsilon$ we obtain from (3.3) the inequality

$$\begin{aligned} \frac{-\varepsilon \cdot 2\mathrm{Re}\left( \zeta \right) -(1-\varepsilon )}{\left|\zeta \right|^2}< \mu _0 < \frac{(1-\varepsilon )\cdot 2\mathrm{Re}\left( \zeta \right) +\varepsilon }{\left|\zeta \right|^2}. \end{aligned}$$

Observing (3.4) yields

$$\begin{aligned} \frac{\varepsilon \left|\zeta -1\right|^2-1}{\left|\zeta \right|^2} -\varepsilon< \mu _0 < \frac{1 - \left|\zeta -1\right|^2 +\varepsilon \left|\zeta -1\right|^2}{\left|\zeta \right|^2} +1-\varepsilon . \end{aligned}$$

Now, (3.5) shows that $\mu _0 \in I_\varepsilon$ and therefore $\zeta ^{-1} z \in D$.

The case $\mathrm{Re}\left( \zeta \right) \le 0$ can be proven similarly. Here we have

$$\begin{aligned} \left|\zeta -1\right|^2-\left|\zeta \right|^2 \le \varepsilon \left|\zeta -1\right|^2 < \left|\zeta \right|^2 \end{aligned}$$

and (3.3) as well as (3.4) yield the estimation

$$\begin{aligned}&\frac{\varepsilon \left|\zeta -1\right|^2-\left|\zeta -1\right|^2}{\left|\zeta \right|^2} + 1-\varepsilon = \frac{(1-\varepsilon ) (2\mathrm{Re}\left( \zeta \right) -1)}{\left|\zeta \right|^2} \\&\quad< \mu _0 < \frac{\varepsilon (1- 2\mathrm{Re}\left( \zeta \right) )}{\left|\zeta \right|^2} = \frac{\varepsilon \left|\zeta -1\right|^2 }{\left|\zeta \right|^2} - \varepsilon . \end{aligned}$$

From this we again conclude that $-\varepsilon \le \mu _0 < 1-\varepsilon$.

Now we turn to the other direction and show that if $\varepsilon$ is not contained in the stated interval then there exists a $z=\mu _1 (-\overline{\zeta }) + \mu _2 \in D$ such that

$$\begin{aligned}\mu _0=\frac{2\mathrm{Re}\left( \zeta \right) \mu _1- \mu _2}{\left|\zeta \right|^2} \not \in I_\varepsilon \end{aligned}$$

and, hence, $\zeta ^{-1} z \not \in D$. Again we start with $\mathrm{Re}\left( \zeta \right) > 0$.

- Case 1. $0 \le \varepsilon < \nicefrac {1}{\left|\zeta -1\right|^2}$: We choose $\mu _1:=-\varepsilon$ and $\mu _2 \in (\varepsilon \left|\zeta -1\right|^2-\varepsilon , 1-\varepsilon )$. Observe that our assumption on $\varepsilon$ implies that $0 \le \varepsilon \left|\zeta -1\right|^2 <1$, hence our choice of $\mu _2$ is meaningful and $z \in D$. Observing (3.4) yields
  $$\begin{aligned} \mu _0 = \frac{-\varepsilon \cdot 2\mathrm{Re}\left( \zeta \right) -\mu _2}{\left|\zeta \right|^2} < \frac{-\varepsilon \cdot 2\mathrm{Re}\left( \zeta \right) -\varepsilon (\left|\zeta -1\right|^2-1)}{\left|\zeta \right|^2} =-\varepsilon . \end{aligned}$$
- Case 2. $1-\nicefrac {1}{\left|\zeta -1\right|^2}< \varepsilon < 1$: Let $\delta :=1-(1-\varepsilon )\left|\zeta -1\right|^2$ and observe that the assumption on $\varepsilon$ implies $\delta$ to be strictly positive. Since $\mathrm{Re}\left( \zeta \right) >0$ we can choose $\mu _1 \in I_\varepsilon$ such that $\mu _1 > 1-\varepsilon -\delta (2\mathrm{Re}\left( \zeta \right) )^{-1}$. Additionally we let $\mu _2:=-\varepsilon$. Then we have
  $$\begin{aligned} \mu _0 = \frac{\mu _1 \cdot 2\mathrm{Re}\left( \zeta \right) -\mu _2}{\left|\zeta \right|^{2}} > \frac{(1-\varepsilon )\cdot 2\mathrm{Re}\left( \zeta \right) -\delta +\varepsilon }{\left|\zeta \right|^{2}}. \end{aligned}$$
  As before we use (3.4) in order to obtain
  $$\begin{aligned} \mu _0 > 1-\varepsilon + \frac{1 -(1-\varepsilon )\left|\zeta -1\right|^2-\delta }{\left|\zeta \right|^{2}}=1-\varepsilon . \end{aligned}$$

Now we suppose that $\mathrm{Re}(\zeta ) \le 0$. Again we have two cases.

- Case 1. $0 \le \varepsilon < 1-\nicefrac {\left|\zeta \right|^2}{\left|\zeta -1\right|^2}$: Let $\delta :=(1-\varepsilon )\left|\zeta -1\right|^2-\left|\zeta \right|^2$, which is a strictly positive number by the assumption on $\varepsilon$. Set $\kappa :=\delta (1-2\mathrm{Re}\left( \zeta \right) )^{-1} >0$ and choose $\mu _1=\mu _2 \in (1-\varepsilon -\kappa , 1-\varepsilon )$. We use (3.4) and the definitions of $\kappa$ as well as $\delta$ in order to estimate
  $$\begin{aligned} \mu _0= & {} \frac{\mu _1 \cdot 2\mathrm{Re}\left( \zeta \right) -\mu _2}{\left|\zeta \right|^2} \\< & {} \frac{(1-\varepsilon -\kappa )(2\mathrm{Re}\left( \zeta \right) -1)}{\left|\zeta \right|^2} = \frac{\delta +(1-\varepsilon )(\left|\zeta \right|^2-\left|\zeta -1\right|^2)}{\left|\zeta \right|^2} =-\varepsilon . \end{aligned}$$
- Case 2. $\nicefrac {\left|\zeta \right|^2}{\left|\zeta -1\right|^2} \le \varepsilon < 1$: Let $\mu _1=\mu _2=-\varepsilon$. Then
  $$\begin{aligned} \mu _0 = \frac{\mu _1 \cdot 2\mathrm{Re}\left( \zeta \right) -\mu _2}{\left|\zeta \right|^{2}} = \frac{\varepsilon (1-2\mathrm{Re}\left( \zeta \right) )}{\left|\zeta \right|^2} \\ = \frac{\varepsilon (\left|\zeta -1\right|^{2}-\left|\zeta \right|^2)}{\left|\zeta \right|^2} =-\varepsilon +\varepsilon \frac{\left|\zeta -1\right|^{2}}{\left|\zeta \right|^2}\ge 1-\varepsilon . \end{aligned}$$

$$\square $$

With the aid of Proposition 3 we can determine suitable choices for $\varepsilon$ for a given base $\zeta$ such that Condition (3.1) is fulfilled. The following result characterises the bases $\zeta$ for a given $\varepsilon \in [0,1)$.

### Corollary 3

Let $\varepsilon \in (0,1)$. Then Condition (3.1) is satisfied if and only if

### Proof

Suppose at first that $\zeta$ has a positive real part. Then Condition (3.1) is satisfied if and only if $\frac{1}{\left|\zeta -1\right|^2} \le \varepsilon \le 1-\frac{1}{\left|\zeta -1\right|^2}$. This immediately yields the first part of the condition.

If the real part of $\zeta$ is not positive then Condition (3.1) is satisfied if and only if $1-\left|\zeta \right|^2\left|\zeta -1\right|^{-2} \le \varepsilon < \left|\zeta \right|^2\left|\zeta -1\right|^{-2}$. Basic calculations involving (3.4) yield the second part of the condition. $\square$

From this result we immediately see that the case $\varepsilon =\nicefrac {1}{2}$ is the most universal one. Figure 3 in the introduction visualises the statement of Corollary 3 for two choices of $\varepsilon$.

### Example 3

Our setting from Example 1, $\zeta =-\nicefrac {3}{2}+\nicefrac {3\sqrt{3}i}{2}$ and $\varepsilon =\nicefrac {1}{2}$, satisfies Condition (3.1) since $\left|\zeta \right|^2=9$, $\left|\zeta -1\right|^2=14$ and, hence $\varepsilon = \nicefrac {1}{2} \in \left[ \nicefrac {5}{14}, \nicefrac {9}{14}\right)$ (*cf*. Proposition 3). Therefore, we can represent the entire complex plane by zeta-expansions with respect to $\zeta$. Since $\mathbf {d}_{\zeta ,\varepsilon }(\zeta ^{-1}) = 1,(0)^\omega$ we clearly have $(\zeta ^{-1})_{\zeta ,\varepsilon }=0\bullet 1\dot{0} = 0\bullet 100\cdots$, $(1)_{\zeta ,\varepsilon }=1\bullet \dot{0}$ and $(\zeta )_{\zeta ,\varepsilon }=10\bullet \dot{0}$. To obtain $(\overline{\zeta })_{\zeta ,\varepsilon }$ note that $\overline{\zeta }=\nicefrac {\zeta ^{2}}{3}$. In Example 1 we already calculated $\mathbf {d}_{\zeta ,\varepsilon }(\nicefrac {1}{3})$ and from this we immediately obtain that $(\overline{\zeta })_\zeta = (-1)(-3)\bullet \dot{0}$. Table 3 shows the zeta-expansions for further examples.

**Table 3:** Zeta-expansions with respect to $\zeta =3e^{-\nicefrac {2\pi i}{3}}$ and $\varepsilon =\nicefrac {1}{2}$ for different choices of *z* (Example 3)

*z*	${\varvec{\psi }}_{\zeta }(z)$	$(z)_{\zeta ,\varepsilon }$
$\nicefrac {\overline{\zeta }}{3}$	$(-\nicefrac {1}{3},0)$	$0\bullet 3 \dot{0}$
$\nicefrac {-\zeta }{9}$	$(-\nicefrac {1}{9},\nicefrac {1}{3})$	$0\bullet 0(-3) \dot{0}$
$\left( \zeta -1\right) ^{-1}$	$(-\nicefrac {1}{13},-\nicefrac {1}{13})$	$0\bullet \dot{1}$
$6\left( 1-\zeta \right) ^{-1}$	$(\nicefrac {6}{13},\nicefrac {6}{13})$	$0\bullet (\dot{-6})$
$\left( \zeta +1\right) ^{-1}$	$(-\nicefrac {1}{7},\nicefrac {1}{7})$	$0\bullet \overline{1(-1)}$
$3\left( -\zeta -1\right) ^{-1}$	$(\nicefrac {3}{7},-\nicefrac {3}{7})$	$0\bullet \overline{3(-3)}$
$-\nicefrac {1}{2}$	$(0,-\nicefrac {1}{2})$	$0\bullet {\overline{265}}$
$\nicefrac {\overline{\zeta }}{2}$	$(-\nicefrac {1}{2},0)$	$0\bullet {\overline{526}}$
$\nicefrac {(\overline{\zeta }-1)}{2}$	$(-\nicefrac {1}{2},-\nicefrac {1}{2})$	$0\bullet {\overline{652}}$
$\nicefrac {(1-\overline{\zeta })}{2}$	$(\nicefrac {1}{2},\nicefrac {1}{2})$	$14\bullet {\overline{652}}$

Observe that if the zeta-expansion of *z* has no fractional part then *z* is necessarily an Eisenstein integer. The converse is not true since $\nicefrac {\zeta }{3}$ is also an Eisenstein integer but $(\nicefrac {\zeta }{3})_\zeta = (-1)\bullet (-3)\dot{0}$. In fact, all Eisenstein integers have no fractional part or they terminate with either $\bullet (-3)\dot{0}$ or $\bullet 3\dot{0}$.

### Example 4

Our setting $\zeta =1-i$ and $\varepsilon =\nicefrac {1}{2}$ from Example 2 does not satisfy Condition 3.1 since $\left|\zeta -1\right|=1$ (see Proposition 3). In Fig. 2 we can also see that $D \not \subset \zeta \cdot D$. Therefore, the definition of a zeta-expansion is meaningless due to the lack of uniqueness. Indeed, let $z:=\nicefrac {3}{4}+\nicefrac {i}{2}$ and observe that $z\zeta ^{-3}=-\nicefrac {5}{16}+\nicefrac {i}{16}$. Then from Table 2 we see that *z* has the two different expansions

$$\begin{aligned} 0\bullet 2(-1)01\dot{0} = (-1)2(-2)\bullet 2(-1)01\dot{0}. \end{aligned}$$

## Multiples of roots of unity and relations with real expansions

In the present section we consider the case that the base $\zeta$ is a multiple of certain roots of unity. Here we will be able to show parallels to radix representations with respect to real bases. For this reason we quickly introduce the most important notations and facts.

Consider a real number $\beta$ and an $\varepsilon \in [0,1)$. We define the beta-transformation $T_{\beta ,\varepsilon }$ by

$$\begin{aligned} T_{\beta ,\varepsilon }: I_\varepsilon \longmapsto I_\varepsilon , x \longmapsto \left\{ \beta x\right\} _\varepsilon . \end{aligned}$$

By successive application of $T_{\beta ,\varepsilon }$ we obtain for each real number $x \in I_\varepsilon$ the sequence ${\mathbf{e }}_{\beta ,\varepsilon }(x)=(e_n)_{n \ge 1}$ with $e_n=\left\lfloor \beta T^{n-1}(x) \right\rfloor _\varepsilon$. We immediately see that $e_n \in {{\mathcal {N}}}_{\beta ,\varepsilon }$ with

$$\begin{aligned} {{\mathcal {N}}}_{\beta ,\varepsilon }= {\left\{ \begin{array}{ll} \big (\varepsilon (1-\beta )-1, (\varepsilon -1)(1-\beta )+1\big ) \cap {{\mathbb {Z}}}&{} \text { if } \beta >0, \\ \big ((\varepsilon -1)(1-\beta ), -\varepsilon (1-\beta )\big ] \cap {{\mathbb {Z}}}&{} \text { if } \beta <0. \end{array}\right. } \end{aligned}$$

Now suppose that $\left|\beta \right|>1$. If $x \in I_\varepsilon$ and $\mathbf{e }_{\beta ,\varepsilon }(x)=(e_n)_{n \ge 1}$ then we can represent *x* with respect to the base $\beta$ as $x = \sum _{n \ge 1} e_n \beta ^{-n}$. This representation is known as *beta-expansion*. For positive bases and $\varepsilon =0$ this is a direct generalisation of *q*-ary representations and has been introduced by Rényi [49]. An overview over this well-studied topic can be obtained from [16].

The case $\varepsilon =\nicefrac {1}{2}$, known as the symmetric beta-expansion, is studied in [6] and also covers the balanced ternary notation discussed in [35].

The first research on negative (integer) bases seems to be [26]. More general researches on representations with respect to negative bases are much more recent. In [31] the case $\varepsilon =\beta (\beta -1)^{-1}$ was considered. The more general definition from above with arbitrary $\varepsilon \in [0,1)$ can be found in [15].

In order to obtain unique representations we again have to ensure that $\beta ^{-1} I_\varepsilon \subset I_\varepsilon$ (*cf*. Condition (3.1)). While for positive bases this condition is satisfied for all choices of $\varepsilon$, we have to require $\varepsilon \in [(\beta +1)^{-1}, \beta (\beta +1)^{-1})$ for a negative base $\beta$ (see [15, Remark 6]).

In accordance with our notations we say that an integer sequence $(e_n)_{n\ge 1}$ is $(\beta ,\varepsilon )$-admissible if there exists an $x \in I_\varepsilon$ such that $\mathbf{e }_{\beta ,\varepsilon }(x)=(e_n)_{n\ge 1}$. If $\left|\beta \right|>1$ then we have

4.1 $$\begin{aligned} (e_n)_{n\ge 1} \text { is }(\beta ,\varepsilon )\text {-admissible } \Longleftrightarrow \forall m \ge 0: \sum _{n \ge 1} e_{n+m} \beta ^{-n} \in I_\varepsilon . \end{aligned}$$

Admissible sequences with respect to real bases can also be characterised in terms of two reference sequences. Recall that an integer sequence $(e_n)_{n \ge 1}$ is lexicographically smaller than another integer sequence $(e'_n)_{n \ge 1}$ if $e_{n_0} < e'_{n_0}$, where $n_0 \ge 1$ is the smallest index such that $e_{n_0} \not = e'_{n_0}$. In this case we write $(e_n)_{n \ge 1} <_{\mathrm{lex}} (e'_n)_{n \ge 1}$. We say hat $(e_n)_{n \ge 1}$ is smaller than $(e'_n)_{n \ge 1}$ with respect to the alternating order, and write $(e_n)_{n \ge 1} <_{\mathrm{alt}} (e'_n)_{n \ge 1}$, if $(-1)^{n_0} e_{n_0} < (-1)^{n_0}e'_{n_0}$ and $n_0 \ge 1$ is the smallest index such that $e_{n_0} \not = e'_{n_0}$. Now, there exist particular (characteristic) integer sequences $\mathbf {e}^*(-\varepsilon )$ and $\mathbf {e}^*(1-\varepsilon )$ such that

$$\begin{aligned}&(d_n)_{n \ge 1} \text { is }(\beta ,\varepsilon )\hbox {-admissible } \\&\quad \Longleftrightarrow \forall m \ge 0: {\left\{ \begin{array}{ll} \mathbf {e}^*(-\varepsilon ) \le _{\mathrm{lex}} (e_{n+m})_{n \ge 1}<_{\mathrm{lex}} \mathbf {e}^*(1-\varepsilon ) &{} \text { if } \beta >1, \\ \mathbf {e}^*(-\varepsilon ) \le _{\mathrm{alt}} (e_{n+m})_{n \ge 1}<_{\mathrm{alt}} \mathbf {e}^*(1-\varepsilon ) &{} \text { if } \beta <-1. \end{array}\right. } \end{aligned}$$

For the exact shape of these characteristic sequences $\mathbf {e}^*(-\varepsilon )$ and $\mathbf {e}^*(1-\varepsilon )$ we refer to [6, 15, 31, 32, 45].

The $(\beta ,\varepsilon )$-admissible sequences induce a subshift $\Omega _{\beta ,\varepsilon }$ known as beta-shift. It is defined in a straightforward way as

$$\begin{aligned} \Omega _{\beta ,\varepsilon }:= \overline{\{\mathbf {e}_{\beta ,\varepsilon }(z) : x \in I_\varepsilon \}}. \end{aligned}$$

Note that $\Omega _{\beta ,\varepsilon }$ is sofic if and only if the characteristic sequences $\mathbf {e}^*(-\varepsilon )$ and $\mathbf {e}^*(1-\varepsilon )$ are eventually periodic.

Observe that a radix representation with respect to a negative base can be easily converted into a representation with respect to a positive base by observing that

$$\begin{aligned} \sum _{n \ge 1} e_{n}(-\beta )^{-n} = \sum _{n \ge 1} (-\beta e_{2n-1} + e_{2n})(\beta ^2)^{-n}. \end{aligned}$$

This idea was used in [28] to study (lexicographically) extremal representations with respect to a negative base. For integer bases these considerations yield a relation between the admissibility with respect to different bases.

### Proposition 4

Let $\beta =N \in {{\mathbb {Z}}}\setminus \{-1,0,1\}$ and $\varepsilon \in [0,1)$. A sequence $(e_n)_{n \ge 1}$ of bounded integers is $(N,\varepsilon )$-admissible if and only if $(Ne_{2n-1}+e_{2n})_{n\ge 1}$ and $(Ne_{2n}+e_{2n+1})_{n \ge 1}$ are $(N^2,\varepsilon )$-admissible.

The proof uses (4.1) and is straightforward. Observe that the boundedness of the integers is necessary for ensuring convergence. Indeed, without this requirement the proposition does not hold. As a counterexample consider the alternating exponential sequence $((-N)^n)_{n \ge 1}$ which is obviously not $(-N,\varepsilon )$-admissible but $(N \cdot (-N)^{2n-1} + (-N)^{2n})_{n\ge 1} =(N \cdot (-N)^{2n} + (-N)^{2n+1})_{n\ge 1}= (0)^\omega$ is $(N^2,\varepsilon )$-admissible. The following results show analogous relations between complex and real bases.

### Multiples of third and sixth roots of unity

Suppose that $\zeta = N e^{\pm \nicefrac {2\pi i}{3}}$ with $N \not =0$ an integer. Therefore, $\zeta$ is either an integer multiple of a primitive third root of unity (for $N \ge 1$) or an integer multiple of a primitive sixth root of unity (for $N \le -1$). Note that we have $\zeta ^2+N\zeta +N^2=0$ and $\left|\zeta -1\right|^2=1+N+N^2$ Depending on the particular choice of *N* the induced set of digits is given by

$$\begin{aligned} {{\mathcal {N}}}:={\left\{ \begin{array}{ll} \{d \in {{\mathbb {Z}}}: (\varepsilon -1)(N^2+N+1)+N< d< \varepsilon (N^2+N+1) - N\} &{} \text { if } N\ge 2, \\ \{d \in {{\mathbb {Z}}}: (\varepsilon -1)(N^2+N+1) < d \le \varepsilon (N^2+N+1)\} &{} \text { if } N\le -2. \end{array}\right. } \end{aligned}$$

#### Theorem 3

Let $N \in {{\mathbb {Z}}}\setminus \{-1,0,1\}$, $\zeta =N e^{\pm \nicefrac {2\pi i}{3}}$, $\varepsilon \in [0,1)$, and $(d_n)_{n \ge 1}$ be a sequence of bounded integers. Then the following assertions are equivalent.

(i) The sequence $(d_n)_{n \ge 1}$ is $(\zeta ,\varepsilon )$-admissible.
(ii) For each $k \in \{0,1,2\}$ the sequence $(e^{(k)}_{n})_{n \ge 1}$ is $(N^3,\varepsilon )$-admissible, where $e^{(k)}_n:=-Nd_{3n-2+k} +d_{3n-1+k}$.

#### Proof

At first we show that (i) $\Rightarrow$ (ii). Let $z \in D$ such that $\mathbf {d}_{\zeta ,\varepsilon }(z)=(d_n)_{n \ge 1}$. For each integer $n \ge 0$ define $z_n:=S^n(z_0)$ (i.e., $z_0=z$) and $\mu _n =(z_n-\overline{z_n})(\zeta -\overline{\zeta })^{-1}$. Due to Proposition 1 we have $\mu _n \in I_\varepsilon$ and $z_n = -\overline{\zeta }\mu _n + \mu _{n+1}$. By definition we have

$$\begin{aligned}\ S^3(z_{n}) = z_{n+3}= \zeta ^3 z_n - d_{n+1} \zeta ^{2} - d_{n+2} \zeta ^{1} - d_{n+3}. \end{aligned}$$

We subtract the complex conjugate equation, divide by $\zeta -\overline{\zeta }$ and observe that $\zeta ^{3}=\overline{\zeta }^{3} = N^{3}$ in order to obtain

$$\begin{aligned} \mu _{n+3} = N^3 \mu _n - d_{n+1} P_2(\zeta ) - d_{n+2} P_1(\zeta ). \end{aligned}$$

It is easy to see that $d_{n+1} P_2(\zeta ) - d_{n+2} P_1(\zeta ) =-Nd_{n+1} + d_{n+2} \in {{\mathbb {Z}}}$. Since $\mu _{n}$ as well as $\mu _{n+3}$ are contained in $I_\varepsilon$ we conclude that $\mu _{n+3}=T_{N^3,\varepsilon }(\mu _1)$ which immediately induces Item (ii).

On the other hand, assume that Item (ii) holds. From the boundedness of the members of $(d_n)_{n \ge 1}$ we conclude that $\sum _{n \ge 1} d_{n+m}\zeta ^{-n}$ converges for all $m \ge 1$ and, hence, $\mu _{m}:=\sum _{n \ge 1} d_{m+n} P_{-n}(\zeta )$ is well defined. Now observe that for each $n \in {{\mathbb {Z}}}$ we have

$$\begin{aligned} P_n(\zeta ):=\frac{\zeta ^{n}-\overline{\zeta ^{n}}}{\zeta -\overline{\zeta }} = N^{n-1}\frac{\sin (\nicefrac {2n\pi }{3})}{\sin (\nicefrac {2\pi }{3})} = {\left\{ \begin{array}{ll} 0 &{} \text { if } n \equiv 0 \, (\mathrm{mod\,}3), \\ N^{n-1} &{} \text { if } n \equiv 1 \, (\mathrm{mod\,}3), \\ -N^{n-1} &{} \text { if } n \equiv 2 \, (\mathrm{mod\,}3). \end{array}\right. } \end{aligned}$$

From this we immediately obtain $\mu _{3m+k}=\sum _{n \ge 1} e^{(k)}_{n+m} N^{-3n}$ and since $(e^{(k)}_{n})_{n \ge 1}$ is $(N^3,\varepsilon )$-admissible we conclude that $\mu _{3m+k} \in I_\varepsilon$. Now the assertion follows immediately from Theorem 1. $\square$

With this theorem we can characterise the zeta-shift $\Omega _{\zeta ,\varepsilon }$ for bases of the discussed shape.

#### Corollary 4

With the notations from Theorem 3 we have that a sequence $(d_n)_{n \ge 1}$ of bounded integers is contained in $\Omega _{{\zeta ,\varepsilon }}$ if and only if $(e^{(k)}_{n})_{n \ge 1} \in \Omega _{N^3,\varepsilon }$ for each $k \in \{0,1,2\}$ where $e^{(k)}_n:=-Nd_{3n-2+k} +d_{3n-1+k}$.

#### Example 5

The latter results show us some remarkable aspects of zeta-expansions. We want to demonstrate this by the setting $\zeta =3e^{\nicefrac {2\pi }{3}}$ and $\varepsilon =\nicefrac {1}{2}$ (see Example 1 and Example 3). The conditions of Theorem 3 are satisfied and there is a relation with real expansions induced by $(27,\nicefrac {1}{2})$: a digit sequence $(d_n)_{n \ge 1}$ is $(\zeta ,\nicefrac {1}{2})$-admissible if and only if $(e^{(k)}_n)_{n \ge 1}$ is $(27,\nicefrac {1}{2})$-admissible for each $k \in \{0,1,2\}$ where $e^{(k)}_n:=-3d_{3n-2+k} +d_{3n-1+k}$ for all $n \ge 1$.

By [6] the integer sequence $(e_n)_{n \ge 1}$ is $(27,\nicefrac {1}{2})$-admissible if and only if

$$\begin{aligned} \forall m \ge 0 : \mathbf {e}_{27,\nicefrac {1}{2}}(-\nicefrac {1}{2}) \le _{\mathrm{lex}} (d_{n+m})_{n \ge 1} <_{\mathrm{lex}} -\mathbf {e}_{27,\nicefrac {1}{2}}(-\nicefrac {1}{2}). \end{aligned}$$

One easily calculates the characteristic sequence $\mathbf {e}^*(-\nicefrac {1}{2}) = (13)^\omega$, hence, $(e^{(k)}_n)_{n \ge 1}$ is $(27,\nicefrac {1}{2})$-admissible if and only if $-13 \le e^{(k)}_n \le 13$ for all $n \ge 1$ with the restriction that for each $m \ge 0$ we have $(e^{(k)}_{m+n})_{n \ge 1} \not =(13)^\omega$. The latter condition provides the uniqueness. Indeed, we have $\sum _{n \ge 1} 13\cdot (27)^{-n} = 1 +\sum _{n \ge 1} (-13)\cdot (27)^{-n}$ which implies that if for a real number $x \in I_\varepsilon$ the sequence $\mathbf {e}_{27,\nicefrac {1}{2}}(x)$ ends up with $(-13)^\omega$ then there exists another digit sequence (over the alphabet $\{-13, \ldots , 13\}$) that represents *x* (that ends up in $(13)^\omega$).

For our zeta-expansions these observations mean that for each $z \in D$ the sequence $\mathbf {d}_{\zeta ,\nicefrac {1}{2}}(z)$ is characterised by the property that for two consecutive digits $d_n, d_{n+1}$ we always have $-13 \le -3d_n + d_{n+1} \le 13$ and the additional condition

4.2 $$\begin{aligned} \forall m \ge 0: (-3d_{3n+1+m}+d_{3n+2+m})_{n \ge 1} \not =(13)^\omega . \end{aligned}$$

**Fig. 4:**
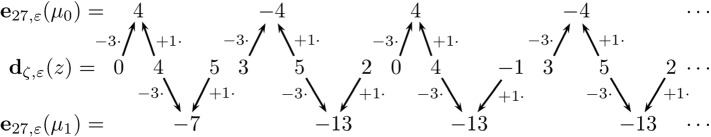
By joining the digits in a correct way we obtain from $\mathbf {d}_{\zeta ,\varepsilon }(z)=0,4,5,(3,5,2,0,4,-1)^\omega$ the two sequences $\mathbf {e}_{27,\varepsilon }(\mu _0)= 4,(-4,4)^\omega$ and $\mathbf {e}_{27,\varepsilon }(\mu _1)=-7,(-13)^\omega$ such that $z=-\overline{\zeta } \mu _0 +\mu _1$

These considerations have several nice consequences. At first, we can obtain from $\mathbf {d}_{\zeta ,\varepsilon }(\mu _0 \overline{\zeta } + \mu _1)$ the sequences $\mathbf {e}_{27,\varepsilon }(\mu _0)$ and $\mathbf {e}_{27,\varepsilon }(\mu _1)$ by recoding. This makes it quite easy to calculate a complex number *z* from its representation. For example, if $\mathbf {d}_{\zeta ,\varepsilon }(z) =0,4,5,(3,5,2,0,4,-1)^\omega$ then we see that $\mu _0=\nicefrac {1}{7}$, $\mu _1=-\nicefrac {5}{18}$ and, hence, $z=-\nicefrac {\overline{\zeta }}7 - \nicefrac {5}{18}$ (see Fig. 4). On the other hand, it is much harder to recover $\mathbf {d}_{\zeta ,\varepsilon }( -\mu _0 \overline{\zeta } + \mu _1)$ directly from the sequences $\mathbf {e}_{27,\varepsilon }(\mu _0)$ and $\mathbf {e}_{27,\varepsilon }(\mu _1)$.

Another interesting observation concerns the complex numbers with more than one expansion. For this reason we consider all digit strings contained in the zeta-shift $\Omega _{\zeta ,\varepsilon }$. In our case the zeta-shift is given by

$$\begin{aligned} \Omega _{\zeta , \nicefrac {1}{2}}= \left\{ (d_n)_{n \ge 1} \in \{-6, \ldots , 6\}^{{\mathbb {N}}}| \forall n \ge 1: -13 \le -3d_n + d_{n+1} \le 13\right\} . \end{aligned}$$

It is obvious that digit strings that violate (4.2) characterise the elements that possess more than one expansion. For example, we easily see from Table 3 that $0\bullet {\overline{265}} = (-1)\bullet \overline{(-2)(-6)(-5)}$; both sequences$-1, (-2,-6,-5)^\omega$ as well as $(2,6,5)^\omega$ are contained in the zeta-shift but the latter one violates Condition (4.2). Similarly, $4 5 \bullet \overline{35204(-1)} = 4 4 \bullet \overline{0(-4)1(-3)(-5)(-2)}$ (see the example from above). We even have four expansions for $\nicefrac {(\zeta +3)}{2}$:

$$\begin{aligned} 0\bullet \overline{(-3)4(-1)} = 12\bullet \overline{3(-4)1} = (-1)\bullet \overline{(-6)(-5)(-2)} = 13\bullet {\overline{652}} \end{aligned}$$

where only the last one is derived from an admissible sequence.

In the associated real $(27,\varepsilon )$ setting two expansions coincide if the digit strings differ by the sequence $1,(-26)^\omega$ (possible filled up by leading zeros). This observation immediately shows (by joining digits) that for our complex zeta-expansions this is performed by the sequence $(1,3,9)^\omega$ (with an appropriate number of zeros at the beginning).

#### Example 6

(*Comparison with Canonical number systems*) Let $\zeta =2e^{\nicefrac {2\pi }{3}}$ and observe that $\left|\zeta \right|^2=4$. It is well known (see for example [33]) that each number $z \in {{\mathbb {C}}}$ can be represented as

4.3 $$\begin{aligned} z = \sum _{n \ge 1} d_n \zeta ^{-n+m} \text { with } d_n \in \{0, \ldots , 3\}. \end{aligned}$$

**Fig. 5:**
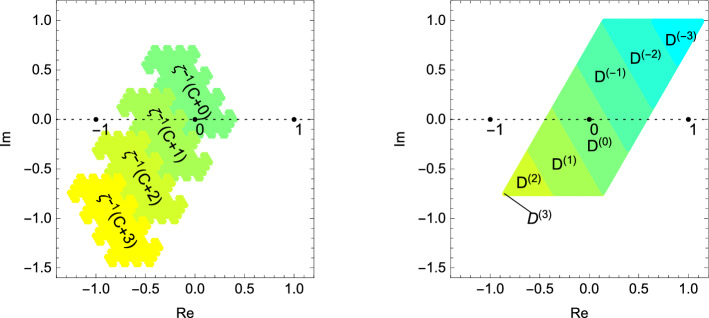
On the left we see the set *C* which is fixed by the set equation $C=\bigcup _{d=0}^3 \zeta ^{-1}(C+d)$. On the right we see the set *D* which is the union of the collection $\{D^{(d)}: d \in \{-3, \ldots , 3\}\}$ that is fixed by graph directed set equations. Observe that $D^{(3)}$ is a singleton

The set

$$\begin{aligned} C:=\left\{ \sum _{n \ge 1} d_n \zeta ^{-n}: (d_n)_{n \ge 1} \in \{0, \ldots , 3\}^{{\mathbb {N}}}\right\} \end{aligned}$$

consists of the complex numbers that can be represented without integer part. This set has a fractal shape (see the left hand side of Fig. 5) and satisfies the set equation $C=\bigcup _{d = 0}^3 \zeta ^{-1} (C+d)$, that is *C* is fixed by an iterated function system (see [29]). The representation (4.3) is unique for almost all complex numbers. The characterisation of elements that possess two or more representations is not that trivial and requires the use of automata (see, for example, [53]). Geometrically this corresponds to a parametrisation of the (fractal) boundary of *C*. For details we refer to respective literature as, for example, [5, 7, 30].

By Proposition 3 the pair (${\zeta ,\varepsilon }$) satisfies (3.1) if $\varepsilon \in [\nicefrac {3}{7}, \nicefrac {4}{7})$. We choose $\varepsilon :=\nicefrac {3}{7}$ which induces the set of digits ${{\mathcal {N}}}=\{-3, \ldots , 3\}$. By Theorem 3 an integer sequence $(d_n)_{n \ge 1}$ is (${\zeta ,\varepsilon }$)-admissible if and only if $(-3)^\omega \le _{\mathrm{lex}} (-2d_{3n+m}+d_{3n+m+1})_{n \ge 0} <_{\mathrm{lex}} (4)^\omega$ holds for all $m \ge 1$. The sequence $(d_n)_{n \ge 1}$ is contained in $\Omega _{\zeta ,\varepsilon }$ if and only if $-3 \le -2d_n + d_{n+1} \le 4$ for all $n \ge 1$.

We have

$$\begin{aligned} D=\left\{ \sum _{n \ge 1}\zeta ^{-n} d_n: (d_n)_{n \ge 1} \text { is }({\zeta ,\varepsilon })\hbox {-admissible}\right\} \end{aligned}$$

and since the map that assigns to a digit sequence the corresponding point in the complex plane is continuous we immediately see that

$$\begin{aligned} {\overline{D}}=\left\{ \sum _{n \ge 1}\zeta ^{-n} d_n: (d_n)_{n \ge 1} \in \Omega _{\zeta ,\varepsilon }\right\} . \end{aligned}$$

For each digit $d \in {{\mathcal {N}}}$ we let

$$\begin{aligned} D^{(d)}:=\left\{ \sum _{n \ge 1}\zeta ^{-n} d_n: (d_n)_{n \ge 1} \in \Omega _{\zeta ,\varepsilon }, d_1=d\right\} . \end{aligned}$$

Obviously ${\overline{D}} = \bigcup _{d \in {{\mathcal {N}}}} D^{(d)}$ and we see that for each $d \in {{\mathcal {N}}}$ the set equation

$$\begin{aligned} D^{(d)}=\bigcup _{d' \in \{2d-3, \ldots , 2d+4\} \cap {{\mathcal {N}}}} \zeta ^{-1}\left( D^{(d')}+d\right) \end{aligned}$$

must hold. This shows that the collection $\{D^{(d)}: d \in {{\mathcal {N}}}\}$ (depicted on the right hand side of Fig. 5) is the fixed set list of a graph directed construction in the sense of [42].

When we compare the sets *C* and *D* and the shift spaces beyond we see that the structure of *C* is complicated but the digit sequences are simple (the full shift). For *D* it is the other way round: the digit sequences are more complicated but the set has a quite simple structure and we can easily characterise the boundary points. In particular, if $(d_n)_{n \ge 1} \in \Omega _{{\zeta ,\varepsilon }}$ then $\sum _{i \ge 1} d_n \zeta ^{-n} \in \partial D$ if and only if one of the four conditions hold:

(i) $$\quad (-2d_{3n+1}+d_{3n+2})_{n\ge 0} = (-3)^\omega $$,
(ii) $$\quad (-2d_{3n+2}+d_{3n+3})_{n\ge 0} = (4)^\omega $$,
(iii) $$\quad (-2d_{3n+1}+d_{3n+2})_{n\ge 0} = (4)^\omega $$,
(iv) $$\quad (-2d_{3n+2}+d_{3n+3})_{n\ge 0} = (-3)^\omega $$.

The four conditions correspond to the four sides of the parallelepiped *D* (counterclockwisely, starting from the bottom).

Note that from Proposition 2 we already know that $\Omega _{\zeta ,\varepsilon }$ is sofic. In fact, we see that it is even of finite type since we can immediately identify

$$\begin{aligned} W_{\Omega _{\zeta ,\varepsilon }}:=\{(-3)d: d \ge -1\} \cup \{(-2)d: d \ge 1\} \cup \{(-1)3\} \\ \cup \{1d: d \le -2\} \cup \{2d: d \le 0\} \cup \{3d: d \le -2\} \end{aligned}$$

as a finite set of forbidden words ($\left|W_{\Omega _{\zeta ,\varepsilon }}\right|=21$).

### Multiples of fourth roots of unity

Suppose that $\zeta =\pm \beta i$ with $\beta >1$ a real number. The induced set of digits is given by

$$\begin{aligned} {{\mathcal {N}}}:=\{d \in {{\mathbb {Z}}}: -(1-\varepsilon )(N^2+1) < d \le \varepsilon (N^2+1)\}. \end{aligned}$$

The following relation is quite obvious and has already been observed in [35] (in a more restricted context).

#### Theorem 4

Let $\beta > 1$ be a real number, $\zeta =\pm \beta i$, $\varepsilon \in [0,1)$ and $(d_n)_{n \ge 1}$ an integer sequence. Then the following assertions are equivalent.

(i) The sequence $(d_n)_{n \ge 1}$ is $({\zeta ,\varepsilon })$-admissible.
(ii) The sequences $\left( d_{2n-1}\right) _{n \ge 1}$ as well as $\left( d_{2n}\right) _{n \ge 1}$ are $(-\beta ^2,\varepsilon )$-admissible.

#### Proof

By Proposition 1 the sequence $(d_n)_{n \ge 1}$ is $({\zeta ,\varepsilon })$-admissible if and only if

$$\begin{aligned} \mu _{m}:=\sum _{n \ge 1}d_{n+m}P_{-n}(\zeta ) \in I_\varepsilon \end{aligned}$$

holds for all $m \ge 0$. For each $n \in {{\mathbb {Z}}}$ we have

$$\begin{aligned} P_{n}(\zeta )=\frac{\zeta ^{n}-\overline{\zeta ^{n}}}{\zeta -\overline{\zeta }} = {\left\{ \begin{array}{ll} 0 &{} \text { if } n \equiv 0 \, (\mathrm{mod\,}2), \\ \beta ^{n-1} &{} \text { if } n \equiv 1 \, (\mathrm{mod\,}4), \\ -\beta ^{n-1} &{} \text { if } n \equiv 3 \, (\mathrm{mod\,}4). \end{array}\right. } \end{aligned}$$

Hence, $\mu _{m}:=\sum _{n \ge 1}(-\beta ^2)^{-n} d_{2n+m-1} \in I_\varepsilon$ for all $m \ge 0$ which is equivalent to the $(-\beta ^2,\varepsilon )$-admissibility of $\left( d_{2n-1}\right) _{n \ge 1}$ and $\left( d_{2n}\right) _{n \ge 1}$. $\square$

### Multiples of eighth roots of unity

Finally we consider the case that $\zeta$ is a certain multiple of a primitive eighth root of unity. In particular, let $\zeta =\sqrt{2}Ne^{\pm \nicefrac {\pi i}{4}}=N \pm Ni$ with $N \in {{\mathbb {Z}}}\setminus \{0\}$. We have $\zeta ^2-2N\zeta +2N^2=0$, hence $\left|\zeta -1\right|^2=1-2N+2N^2$. This yields the set of digits

$$\begin{aligned} {{\mathcal {N}}}:={\left\{ \begin{array}{ll} \left( (\varepsilon -1)(1-2N+2N^2)-2N, \varepsilon (1-2N+2N^2)+2N\right) \cap {{\mathbb {Z}}}&{} \text { if } N \ge 1,\\ \left( (\varepsilon -1)(1-2N+2N^2), \varepsilon (1-2N+2N^2)\right] \cap {{\mathbb {Z}}}&{} \text { if } N \le -1. \end{array}\right. } \end{aligned}$$

#### Theorem 5

Let $N \in {{\mathbb {Z}}}\setminus \{0\}$, $\zeta =\sqrt{2}Ne^{\pm \nicefrac {\pi i}{4}}$, $\varepsilon \in [0,1)$, and $(d_n)_{n \ge 1}$ be a sequence of bounded integers.. Then the following assertions are equivalent.

(i) The sequence $(d_n)_{n \ge 1}$ is $(\zeta ,\varepsilon )$-admissible.
(i) For each $k \in \{0,1,2,3\}$ the sequence $(e^{(k)}_{n})_{n \ge 1}$ is $(-4N^4,\varepsilon )$-admissible, where $e^{(k)}_n:=2N^2d_{4n-3+k} +2Nd_{4n-2+k}+d_{4n-1+k}$.

#### Proof

Observe that for each $n \in {{\mathbb {Z}}}$ we have

$$\begin{aligned} P_n(\zeta ):=\frac{\zeta ^{n}-\overline{\zeta ^{n}}}{\zeta -\overline{\zeta }} = (\sqrt{2}N)^{n-1}\frac{\sin (\nicefrac {n\pi }{4})}{\sin (\nicefrac {\pi }{4})} = {\left\{ \begin{array}{ll} 0 &{} \text { if } n \equiv 0 \, (\mathrm{mod\,}4), \\ \left( \sqrt{2}N\right) ^{n-1} &{} \text { if } n \equiv 1, 3 \, (\mathrm{mod\,}8), \\ \sqrt{2}^n N^{n-1} &{} \text { if } n \equiv 2 \, (\mathrm{mod\,}8), \\ -\left( \sqrt{2}N\right) ^{n-1} &{} \text { if } n \equiv 5, 7 \, (\mathrm{mod\,}8), \\ -\sqrt{2}^n N^{n-1} &{} \text { if } n \equiv 6 \, (\mathrm{mod\,}8). \end{array}\right. } \end{aligned}$$

Then the statement can be shown similarly as Theorem 3. $\square$

#### Example 7

Our case studied in Example 2 ($\zeta =\sqrt{2}e^{-\nicefrac {\pi i}{4}}$ and $\varepsilon =\nicefrac {1}{2}$) satisfies the condition of Theorem 5 (with $N=1$). For this reason we are interested in real expansions with respect to $(-4)$. We have $\mathbf {e}_{-4,\nicefrac {1}{2}}(-\nicefrac {1}{2})=2,(0)^\omega$, hence, a sequence $(e_{n})_{n \ge 1}$ is $(-4,\nicefrac {1}{2})$-admissible if and only if

$$\begin{aligned}\forall m \ge 0: 2,(0)^\omega \le _{\mathrm{alt}} (e_{n+m})_{n \ge 1} <_{\mathrm{alt}} -2,(0)^\omega \end{aligned}$$

(see [15, Theorem 13 and Example 17].) We immediately obtain that a sequence $(d_{n})_{n \ge 1}$ is $(\zeta , \nicefrac {1}{2})$-admissible if and only if

$$\begin{aligned} \forall m \ge 0: 2,(0)^\omega \le _{\mathrm{alt}} (2d_{n+m}+2d_{n+m+1}+d_{n+m+2})_{n \ge 1} <_{\mathrm{alt}} -2,(0)^\omega . \end{aligned}$$

Furthermore, by using Proposition 4 we see that $(d_{n})_{n \ge 1}$ is $(\zeta , \nicefrac {1}{2})$-admissible if and only if

$$\begin{aligned} \forall m \ge 0: -8,(0)^\omega \le _{\mathrm{lex}} (-&8d_{n+m}-8d_{n+m+1}-4d_{n+m+2} \\ +&2d_{n+m+4}+2d_{n+m+5}+d_{n+m+6} )_{n \ge 1} <_{\mathrm{lex}} 8,(0)^\omega \end{aligned}$$

(since $\mathbf {d}_{16,\nicefrac {1}{2}}(-\nicefrac {1}{2})=-8,(0)^\omega$). Therefore we can easily calculate from $\mathbf {d}_{\zeta ,\varepsilon }(-\mu _0\overline{\zeta } + \mu _1)$ the sequences $\mathbf {e}_{-4,\varepsilon }(\mu _0)$ and $\mathbf {e}_{-4,\varepsilon }(\mu _1)$ and, furthermore, $\mathbf {e}_{16,\varepsilon }(\mu _0)$ and $\mathbf {e}_{16,\varepsilon }(\mu _1)$. For example, we immediately see from the last line of Table 2 that $\mathbf {e}_{-4,\nicefrac {1}{2}}(\nicefrac {1}{3}) = (-1,1)^\omega$, $\mathbf {e}_{-4,\nicefrac {1}{2}}(-\nicefrac {1}{15}) = (0,-1)^\omega$, $\mathbf {e}_{16,\nicefrac {1}{2}}(\nicefrac {1}{3}) = (5)^\omega$ and $\mathbf {e}_{16,\nicefrac {1}{2}}(-\nicefrac {1}{15}) = (-1)^\omega$. As we already mentioned in Example 5 it is much more difficult to determine from $\mathbf {e}_{-4,\varepsilon }(\mu _0)$ and $\mathbf {e}_{-4,\varepsilon }(\mu _1)$ or from $\mathbf {e}_{16,\varepsilon }(\mu _0)$ and $\mathbf {e}_{16,\varepsilon }(\mu _1)$ the shape of the sequence $\mathbf {d}_{\zeta ,\varepsilon }(-\mu _0\overline{\zeta } + \mu _1)$.

## Finiteness and periodicity properties

We suppose that $\zeta \in {{\mathbb {C}}}\setminus {{\mathbb {R}}}$ is an algebraic integer and define the sets

$$\begin{aligned} \mathrm{Per}_{\zeta ,\varepsilon }&:=\{z \in D_{\zeta ,\varepsilon }: \exists n \ge 0, k \ge 1: S^n_{\zeta ,\varepsilon }(z)=S^{n+k}_{\zeta ,\varepsilon }(z)\}, \\ \mathrm{Fin}_{\zeta ,\varepsilon }&:=\{z \in D_{\zeta ,\varepsilon }: \exists n \ge 0: S^n_{\zeta ,\varepsilon }(z)=0 \}. \end{aligned}$$

We obviously have $\mathrm{Fin}_{\zeta ,\varepsilon }\subset \mathrm{Per}_{\zeta ,\varepsilon }$. Since $\zeta$ is an algebraic integer we furthermore have $\mathrm{Per}_{\zeta ,\varepsilon }\subseteq {{\mathbb {Q}}}(\zeta )$ and $\mathrm{Fin}_{\zeta ,\varepsilon }\subseteq {{\mathbb {Z}}}[\zeta ^{-1}]$. Motivated by [18] we say that the pair $(\zeta ,\varepsilon )$ possesses the periodicity property (P) (finiteness property (F), respectively) if equality holds, i.e.

P $$\begin{aligned} \mathrm{Per}_{\zeta ,\varepsilon }&= {{\mathbb {Q}}}(\zeta ) \cap D_{\zeta ,\varepsilon }, \end{aligned}$$

F $$\begin{aligned} \mathrm{Fin}_{\zeta ,\varepsilon }&= {{\mathbb {Z}}}[\zeta ^{-1}] \cap D_{\zeta ,\varepsilon }. \end{aligned}$$

In the present section we are going to see that in context with the (P)-property and the (F)-property the complex zeta-expansion behaves analogously to the real beta-expansion.

### Definition 2

Let $\zeta \in {{\mathbb {C}}}\setminus {{\mathbb {R}}}$ be an algebraic integer. We call $\zeta$ a *complex Pisot number* if all algebraic conjugates different from $\zeta$ and $\overline{\zeta }$ are located in the open unit disk. We call $\zeta$ a *complex Salem number* if all algebraic conjugates different from $\zeta$ and $\overline{\zeta }$ are contained in the closed unit disk where at least one such conjugate is located at the unit circle.

While there exists a huge amount of researches concerning real Pisot and Salem numbers (see the surveys [9, 55]) few is published concerning the complex analogues. Algebraic results on complex Pisot numbers can be found in [10, 22, 62]. Some basic properties of complex Salem numbers have been studied in [52] (without using the term *complex Salem number*).

### Remark 1

Let $\zeta$ be a complex Salem number and *P* be its minimal polynomial. Since *P* is irreducible there is actually a pair of complex conjugate roots of modulus 1, hence, *P* is self-reciprocal. This shows that, in fact, *P* has only 4 roots that are not located on the unit circle, namely $\zeta$, $\overline{\zeta }$, $\zeta ^{-1}$ and $\overline{\zeta }^{-1}$. Hence, the degree of *P* is necessarily even and at least 6. Observe that self reciprocal polynomials with roots on the unit circle are studied in [58].

### Example 8

For obtaining complex Salem numbers we can use a strategy proposed in [57]. Let *P* be the minimal polynomial of a complex Pisot number and $n > \deg (P)$. Then $R_{\pm }(t):=t^nP(t) \pm t^{\deg (P)}P(t^{-1})$ has at least $\deg (R_{\pm })-4$ roots of modulus 1. Note that this is a complex version of a result from [51]. For example, let $P(t) = t^3-t^2+1$ and $n=4$. Then

$$\begin{aligned} R_+(t)=t^nP(t) + t^{\deg (P)}P(t^{-1}) = (t^6 - 2 t^5 + 2 t^4 - t^3 + 2 t^2 - 2 t + 1)(t+1) \end{aligned}$$

and one easily verifies that $t^6 - 2 t^5 + 2 t^4 - t^3 + 2 t^2 - 2 t + 1$ is irreducible and has three pairs of complex roots, one outside the unit disk, another one inside the unit disk, and a third one on the unit circle.

Concerning the periodicity property we can state a complex version of the (real) results obtained in [11, 54] that can be shown in an analogue way.

### Proposition 5

Let $\zeta \in {{\mathbb {C}}}$ be an algebraic integer and $\varepsilon \in [0,1)$. If $\zeta$ is a complex Pisot number then $(\zeta ,\varepsilon )$ satisfies (P). On the other hand if $(\zeta ,\varepsilon )$ satisfies (P) then $\zeta$ is a complex Pisot number or complex Salem number.

For characterising pairs $(\zeta ,\varepsilon )$ that have the finiteness property (F) we can use so-called shift radix systems. By shift radix systems we mean a family of ${{\mathbb {Z}}}^d$-actions that are related with Canonical number systems as well as beta-expansions (see [2]). We show that there is an analogue connection with the zeta-transformation.

### Definition 3

Let $\varepsilon \in [0,1)$, $\mathbf {r} = (r_0, \ldots , r_{d-1}) \in {{\mathbb {R}}}^d$ and define the map ${\varvec{\tau }}_{\mathbf {r},\varepsilon }: {{\mathbb {Z}}}^d \longrightarrow {{\mathbb {Z}}}^d$ by

$$\begin{aligned} \begin{aligned} {\varvec{\tau }}_{\mathbf {r},\varepsilon }: \,&{{\mathbb {Z}}}^d \rightarrow {{\mathbb {Z}}}^d, \\&(x_0,\ldots ,x_{d-1}) \mapsto \left( x_1,\ldots ,x_{d-1},-\left\lfloor \sum _{j=0}^{d-1} r_jx_j \right\rfloor _\varepsilon \right) . \end{aligned} \end{aligned}$$

We call the dynamical system $({{\mathbb {Z}}}^d, {\varvec{\tau }}_{\mathbf {r},\varepsilon })$ a *shift radix system* if for each $\mathbf{x } \in {{\mathbb {Z}}}^d$ there exists an $n \in {{\mathbb {N}}}$ such that ${\varvec{\tau }}^n_{\mathbf {r},\varepsilon }({\varvec{x}})={\varvec{0}}$.

Note that shift radix systems have been originally defined in [2] for $\varepsilon =0$ only. Later, the case $\varepsilon =\nicefrac {1}{2}$ has been introduced in [6] as symmetric shift radix systems. The general definition from above was considered in [56]. A survey on shift radix systems can be found in [34].

Now let $\zeta \in {{\mathbb {C}}}\setminus {{\mathbb {R}}}$ be an algebraic integer of degree $d+2$ and denote by $P(t)=t^{d+2}+p_{d+1}t^{d+1}+\ldots +p_0$ its minimal polynomial. Let $s_0, \ldots , s_{d+1}$ and $r_0, \ldots , r_{d}$ such that

$$\begin{aligned}P(t)=(t - \zeta )\cdot \sum _{j=0}^{d+1} s_j t^j = (t - \zeta )\cdot (t - \overline{\zeta }) \cdot \sum _{j=0}^{d} r_j t^j.\end{aligned}$$

Note that $(t-\zeta )(t-\overline{\zeta })=t^2-a_1t-a_0$ with $a_0$ and $a_1$ defined as in Proposition 1. Clearly, $s_{d+1}=r_d=1$ and $r_j \in {{\mathbb {R}}}$ for each $j \in \{0, \ldots , d-1\}$ while in general the $s_j$ are not real numbers. Furthermore, the reader easily verifies the following relations:

5.1 $$\begin{aligned} \begin{array}{ll} s_0 &{} = -\zeta ^{-1}p_0, \\ s_j &{} = \zeta ^{-1}(-p_{j}+ s_{j-1}) \quad (1 \ge j \ge d), \\ s_{d} &{} = p_{d+1} + \zeta , \end{array} \quad \begin{array}{ll} r_{0} &{} = -\overline{\zeta }^{-1}s_0, \\ r_j &{} = \overline{\zeta }^{-1}(-s_{j}+ r_{j-1}) \quad (1 \ge j \ge d-1), \\ r_{d-1} &{} = s_{d} + \overline{\zeta }. \end{array}\nonumber \\ \end{aligned}$$

Now define the function

$$\begin{aligned} \phi _{\mathbf {r},\varepsilon }: {{\mathbb {Z}}}^d \longrightarrow {{\mathbb {C}}}, (x_0,\ldots ,x_{d-1}) \longmapsto \sum _{j=0}^{d} r_j(-\overline{\zeta } x_j + x_{j+1}) \end{aligned}$$

with

$$\begin{aligned} x_d=-\left\lfloor \sum _{j=0}^{d-1} r_j x_j \right\rfloor _\varepsilon , x_{d+1}=-\left\lfloor \sum _{j=0}^{d-1} r_j x_{j+1} \right\rfloor _\varepsilon . \end{aligned}$$

### Lemma 1

The function $\phi _{\mathbf {r},\varepsilon }$ maps ${{\mathbb {Z}}}^d$ bijectively onto ${{\mathbb {Z}}}[\zeta ] \cap D$.

### Proof

At first we show that $\phi _{\mathbf {r},\varepsilon }$ is an injective map and $\phi _{\mathbf {r},\varepsilon }({{\mathbb {Z}}}^d) \subset {{\mathbb {Z}}}[\zeta ] \cap D$. Let $(x_0,\ldots ,x_{d-1}) \in {{\mathbb {Z}}}^d$. Then we have

$$\begin{aligned}&\phi _{\mathbf {r},\varepsilon }(x_0,\ldots ,x_{d-1}) = -\overline{\zeta } \sum _{j=0}^{d} r_j x_j + \sum _{j=0}^{d} r_j x_{j+1} \\&\qquad = -\overline{\zeta } \left( \sum _{j=0}^{d-1} r_j x_j - r_d\left\lfloor \sum _{j=0}^{d-1} r_j x_j \right\rfloor _\varepsilon \right) + \left( \sum _{j=0}^{d-1} r_j x_{j+1} - r_d\left\lfloor \sum _{j=0}^{d-1} r_j x_{j+1} \right\rfloor _\varepsilon \right) . \end{aligned}$$

Since $r_d=1$ we immediately see that $\phi _{\mathbf {r},\varepsilon }(x_0,\ldots ,x_{d-1}) \in D$. By definition and (5.1) we have

5.2 $$\begin{aligned} \phi _{\mathbf {r},\varepsilon }(x_0,\ldots ,x_{d-1}) = -\overline{\zeta } r_0 x_0 + \sum _{j=1}^{d} (-\overline{\zeta }r_{j}+r_{j-1})x_j + r_d x_{d+1} = \sum _{j=0}^{d} s_j x_j. \end{aligned}$$

It is easy to see that $\{s_{d+1}, s_d, \ldots , s_0\}$ is a basis of the ${{\mathbb {Z}}}$-module ${{\mathbb {Z}}}[\zeta ]$, hence, $\phi _{\mathbf {r},\varepsilon }(x_0,\ldots ,x_{d-1}) \in {{\mathbb {Z}}}[\zeta ]$. If $\phi _{\mathbf {r},\varepsilon }(x_0,\ldots ,x_{d-1})=\phi _{\mathbf {r},\varepsilon }(x'_0,\ldots ,x'_{d-1}) \in {{\mathbb {Z}}}^d$ for some $(x'_0,\ldots ,x'_{d-1}) \in {{\mathbb {Z}}}^d$ then (5.2) implies that $x_j=x'_j$ for $j \in \{0,\ldots ,d-1\}$ which yields the injectivity of $\phi _{\mathbf {r},\varepsilon }$.

Now we show the surjectivity. Let $z \in {{\mathbb {Z}}}[\zeta ] \cap D$. Then there exist uniquely determined integers $x_0, \ldots , x_{d+1}$ such that $z=\sum _{j=0}^{d+1} s_j x_j$. From this we easily obtain

$$\begin{aligned}z=-\overline{\zeta } \sum _{j=0}^{d} r_j x_j + \sum _{j=0}^{d} r_j x_{j+1}\end{aligned}$$

by using (5.1). As $z \in D$ we clearly have $\sum _{j=0}^{d} r_j x_j \in I_\varepsilon$ as well as $\sum _{j=0}^{d} r_j x_{j+1} \in I_\varepsilon$ and since $r_d=1$ we obtain

$$\begin{aligned} -\varepsilon \le \sum _{j=0}^{d} r_j x_j=&\sum _{j=0}^{d-1} r_j x_j + x_d< 1-\varepsilon ,\\ -\varepsilon \le \sum _{j=0}^{d} r_j x_{j+1}=&\sum _{j=0}^{d-1} r_j x_{j+1} + x_{d+1} < 1-\varepsilon ,\\ \end{aligned}$$

which immediately proves that $z = \phi _{\mathbf {r},\varepsilon }(x_0,\ldots ,x_{d-1})$. $\square$

### Theorem 6

We have $S\circ \phi _{\mathbf {r},\varepsilon }=\phi _{\mathbf {r},\varepsilon } \circ {\varvec{\tau }}_{\mathbf {r},\varepsilon }$ on ${{\mathbb {Z}}}^d$.

### Proof

Let $\mathbf{x }=(x_0,\ldots ,x_{d-1}) \in {{\mathbb {Z}}}^d$, and set $x_d:=-\left\lfloor \sum _{j=0}^{d-1} r_j x_j \right\rfloor _\varepsilon$, $x_{d+1}:=-\left\lfloor \sum _{j=0}^{d-1} r_j x_{j+1} \right\rfloor _\varepsilon$ and $x_{d+2}:=-\left\lfloor \sum _{j=0}^{d-1} r_j x_{j+2} \right\rfloor _\varepsilon$. By definition and (5.2) we have

$$\begin{aligned} \phi _{\mathbf {r},\varepsilon } \circ {\varvec{\tau }}_{\mathbf {r},\varepsilon } (x_0,\ldots ,x_{d-1}) = \phi _{\mathbf {r},\varepsilon }(x_1,\ldots ,x_{d}) = \sum _{j=0}^{d+1} s_{j}x_{j+1}. \end{aligned}$$

On the other hand,

$$\begin{aligned} S \circ \phi _{\mathbf {r},\varepsilon }(x_0,\ldots ,x_{d-1}) = \zeta \phi _{\mathbf {r},\varepsilon }(x_0,\ldots ,x_{d-1}) -d = \zeta \sum _{j=0}^{d+1} s_{j}x_{j}-d, \end{aligned}$$

with $d\in {{\mathbb {Z}}}$ such that $\zeta \sum _{j=0}^{d+1} s_{j}x_{j}-d \in D$. Note that by (5.1) we have $\zeta s_j = s_{j-1} -p_{j}$ for each $1 \le j \le d+1$ and $\zeta s_0 = -p_0$. Thus,

$$\begin{aligned} \zeta \phi _{\mathbf {r},\varepsilon }(x_0,\ldots ,x_{d-1}) -d = \zeta \sum _{j=0}^{d+1} s_{j}x_{j} -d = \sum _{j=0}^{d} s_{j}x_{j+1} -\sum _{j=0}^{d+1} x_j p_j-d \in D. \end{aligned}$$

Since $\{s_0, \ldots , s_{d}, 1\}$ is a basis of ${{\mathbb {Z}}}[\zeta ]$ and by the injectivity of $\phi _{\mathbf {r},\varepsilon }$ we must have that $-\sum _{j=0}^{d+1} x_j p_j-d =x_{i+2}$, hence,

$$\begin{aligned} S\circ \phi _{\mathbf {r},\varepsilon }(x_0,\ldots ,x_{d-1}) = \phi _{\mathbf {r},\varepsilon }(x_1,\ldots ,x_{d}) = \phi _{\mathbf {r},\varepsilon } \circ {\varvec{\tau }}_{\mathbf {r},\varepsilon } (x_0,\ldots ,x_{d-1}). \end{aligned}$$

$$\square $$

Observe that the latter result generalises [41, Theorem 7.1] and shows that the following commutative diagram holds:

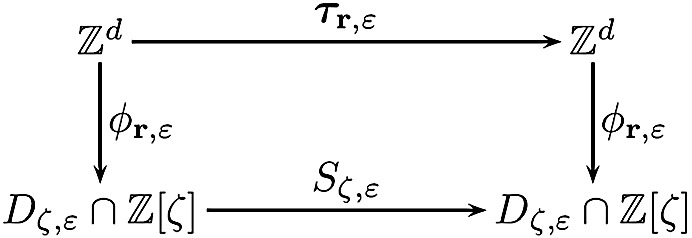



We now easily obtain that bases with the finiteness property can be described by shift radix systems.

### Corollary 5

Let $\zeta$ be a non-real algebraic integer with minimal polynomial

$$\begin{aligned} (t^d + r_{d-1}t^{d-1}+ \cdots +r_0)(t-\zeta )(t-\overline{\zeta }) \end{aligned}$$

and $\varepsilon \in [0,1)$. Then the pair $({\zeta ,\varepsilon })$ satisfies (F) if and only if $({{\mathbb {Z}}}^d,\tau _{\mathbf {r}, \varepsilon })$, with $\mathbf {r}=(r_0, \ldots , r_{d-1})$, is a shift radix system.

### Proof

If $z_0 \in {{\mathbb {Z}}}[\zeta ^{-1}] \cap D$ then there clearly exists an $j \in {{\mathbb {N}}}$ such that $z:=S^j(z_0) \in {{\mathbb {Z}}}[\zeta ] \cap D$. By Theorem 6 we have $S^k(z)=0$ if and only if ${\varvec{\tau }}_{\mathbf {r},\varepsilon }^k\circ \phi _{\mathbf {r},\varepsilon }^{-1}(z) = \mathbf {0}$. Thus, ${{\mathbb {Z}}}[\zeta ^{-1}] \cap D \subset \mathrm{Fin}_{\zeta ,\varepsilon }$ if and only if $({{\mathbb {Z}}}^d,{\varvec{\tau }}^k_{\mathbf {r},\varepsilon })$ is a shift radix system. $\square$

The corollary is a complex version of [2, Theorem 2.1], [6, Theorem 3.7] and [56, Theorem 3.4].

### Example 9

Consider the polynomial $t^4-2t^3+4t^2-2t+1$ and let

$$\begin{aligned} \zeta = \frac{1 + \sqrt{\sqrt{5}-2}}{2} - \frac{1+\sqrt{\sqrt{5}+2}}{2}i \approx 0.7429 - 1.5291 i \end{aligned}$$

be one of its roots (*cf.* right hand side of Fig. 1). The other roots are given by $\overline{\zeta }$, $\zeta ^{-1}$ and $\overline{\zeta }^{-1}$, especially, $\zeta$ is a complex Pisot number. Whether $(\zeta ,\varepsilon )$ satisfies (F) depends on the choice of $\varepsilon$. By Corollary 5 we are interested in the transformation $\tau _{\mathbf {r},\varepsilon }$ induced by $\mathbf {r}=(r_0,r_1)$ such that $(t-\zeta ^{-1})(t-\overline{\zeta }^{-1}) = t^2+r_1t+r_0$, that is

$$\begin{aligned} r_1=&-1+\sqrt{\sqrt{5}-2} \approx -0.5141, \\ r_0=&\frac{1}{2}\left( 1+\sqrt{5} - \sqrt{\sqrt{5}-2} -\sqrt{\sqrt{5}+2}\right) \approx 0.3460. \end{aligned}$$

By [6, Theorem 5.2] the pair $(\zeta ,\nicefrac {1}{2})$ satisfies (F). On the other hand, by [56, Theorem 5.2 and Theorem 5.11], $(\zeta ,\varepsilon )$ does not satisfy (F) if $\varepsilon < -r_0-r_1 \approx 0.1681$ or $\varepsilon \ge 1+r_0+r_1 \approx 0.8319$.
